# Effects of creatine supplementation on muscle strength gains—a meta-analysis and systematic review

**DOI:** 10.7717/peerj.20380

**Published:** 2025-11-27

**Authors:** Haoda Zhang, Tian Lan, Xueru Yan, Haoran Gu, Yanhong Li, Enpeng He

**Affiliations:** 1 Xinjiang Normal University Sports Human Science Laboratory, Xinjiang Normal University, Urumqi, Xinjiang Uygur Autonomous Region, China; 2 College of Life Sciences, Xinjiang Normal University, Urumqi, Xinjiang Uygur Autonomous Region, China

**Keywords:** Creatine (Cr), Phosphocreatine (PCr), Muscle strength, Young people, Middle-aged and elderly people, High-intensity training, Dosage

## Abstract

Creatine (Cr) is a widely utilized nutritional supplement. Empirical evidence indicates that Cr supplementation significantly elevates intramuscular Cr content, thereby providing an energy substrate reservoir for exercise performance and facilitating improvements in muscle strength. Although numerous studies have examined the relationship between Cr and muscle strength, comprehensive investigations into population-specific ergogenic responses, optimal Cr dosage, and concomitant training intensity remain limited. These parameters are critical determinants for maximizing the efficacy of Cr supplementation. Against this backdrop, this study adopts meta-analysis, integrating conventional and Robust Variance Estimation (RVE) models, to comprehensively evaluate the intervention effects of Cr supplementation across three dimensions: population applicability, dosage optimization, and training intensity (PROSPERO registration number: CRD42024547697). It aims to provide evidence-based support for precise clinical application and subsequent research directions.

**Results:**

Results showed no statistical difference in baseline muscle strength between the Cr intervention group and the control group; after intervention, the Cr group exhibited significant strength gains. Further subgroup analysis revealed: untrained individuals had greater muscle strength improvements than trained ones; the low-to-moderate dose group showed better effects than the high-dose group; high-intensity training had more significant effect sizes than low-intensity training; and no definitive conclusion was reached on muscle strength improvements between middle-aged/elderly and young populations.

**Conclusion:**

Cr supplementation significantly improves muscle strength in the general population. Specifically: untrained individuals show greater muscle strength improvements; low-dose supplementation combined with high-intensity exercise yields better effects; no definitive conclusion was reached on effect differences between middle-aged/elderly and young populations, requiring larger-sample studies for more precise effect size analysis.

## Introduction

**Figure 1:**
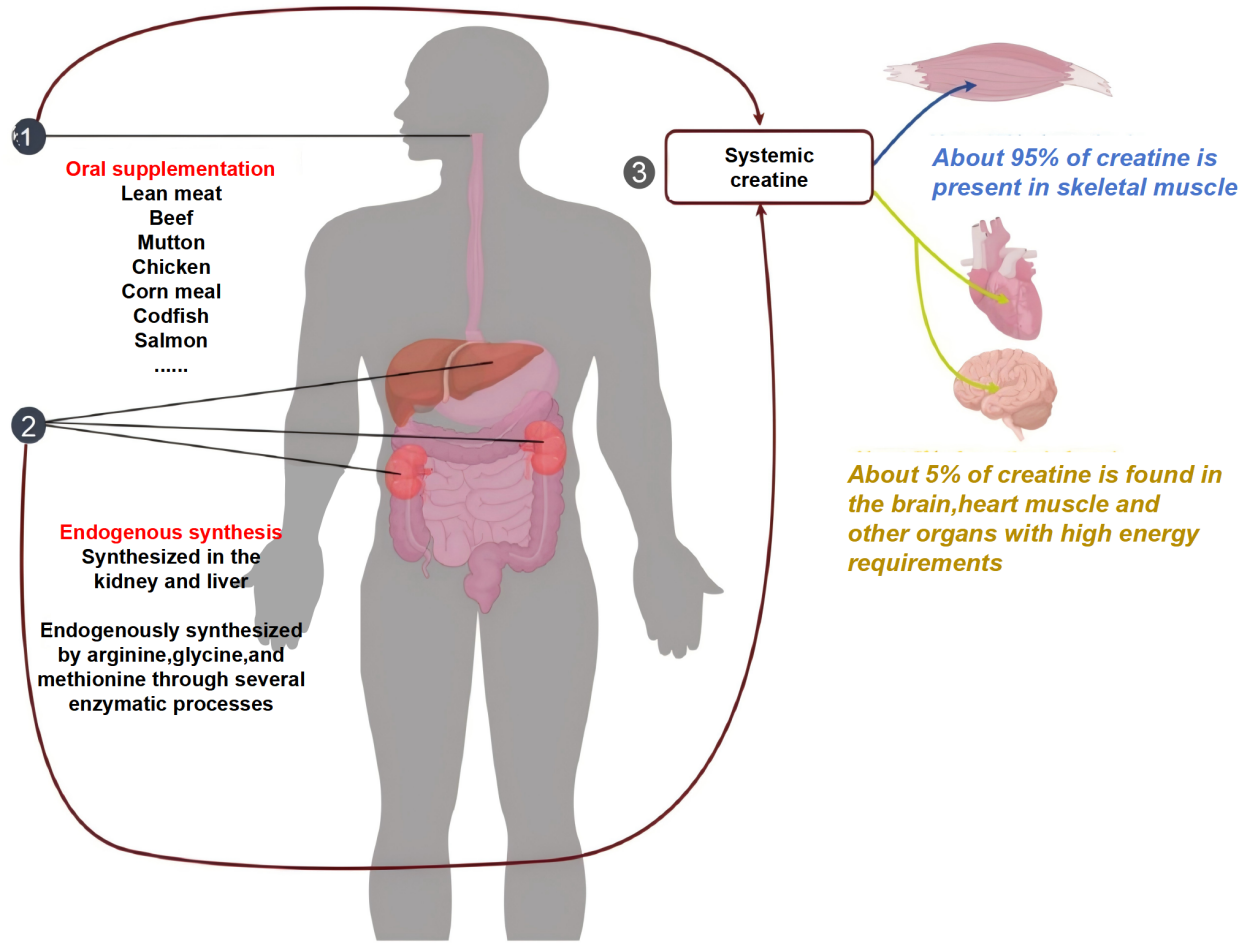
Composition of the total creatine pool in the body and distribution of creatine in the body. ①: Regular diets such as red meat and seafood can provide 60%–80% of creatine and phosphocreatine to the human body. ②: Creatine can also be endogenously synthesized from arginine, glycine, and methionine in the liver and kidneys.③: The majority of creatine (approximately 95%) is present in skeletal muscles, with small amounts in the brain, cardiac muscle, and testes (approximately 5%).

Creatine (Cr) is a naturally occurring non-protein amino acid endogenously synthesized in the liver and kidneys through multi-enzymatic processes involving arginine, glycine, and methionine, predominantly found in red meat and seafood (Suzuki et al., 2004; Bertin et al., 2007; Harris, 2011; Sahlin & Harris, 2011; Kreider & Jung, 2011; Ostojic, 2021). Approximately 95% of bodily Cr resides in skeletal muscle, with the remaining 5% distributed in the brain, myocardium, and testes (Buford et al., 2007; Kreider & Jung, 2011) (Fig. 1). A conventional diet typically provides 60%–80% of Cr and phosphoCreatine (PCr) stores, while exogenous Cr supplementation can further elevate muscular Cr/PCr concentrations by 20–40% (Harris, Söderlund & Hultman, 1992; Hultman et al., 1996; Kreider et al., 2017). Substantial evidence confirms that Cr supplementation exerts positive effects on muscle strength enhancement across the general population (Smith et al., 1998; Lamontagne-Lacasse, Nadon & Goulet, 2011; Zuniga et al., 2012; Cooper et al., 2012; Claudino et al., 2014; Lanhers et al., 2017; Fernández-Landa et al., 2019).

Muscle strength refers to the ability of muscle fibers to generate contraction through the sliding of myofilaments, thereby overcoming external resistance. This process involves a series of energy conversions, with the chemical energy derived primarily from ATP hydrolysis being critical. The propagation of excitatory action potentials triggers an elevation in sarcoplasmic Ca^2^^+^ concentration, enabling Ca^2^^+^ to bind to troponin C (TnC). This binding subsequently releases the inhibitory effect of troponin I (TnI) on myosin, permitting the formation of myosin cross-bridges with actin. Following this, energy released from ATP hydrolysis on myosin drives the sliding of actin filaments from the I band toward the A band, resulting in sarcomere shortening and muscle fiber contraction (Marston & Zamora, 2020). When action potentials cease, the sarcoplasmic/endoplasmic reticulum Ca^2^^+^-ATPase (SERCA) actively transports Ca^2^^+^ from the sarcoplasm back into the sarcoplasmic reticulum against its concentration gradient, consuming ATP in the process. This restores Ca^2^^+^ homeostasis within the muscle fiber and prepares it for subsequent contractions. Concurrently, the decline in cytoplasmic Ca^2^^+^ concentration causes dissociation of Ca^2^^+^ from troponin C. Consequently, the linkage between actin and myosin cross-bridges is severed, allowing the sarcomere to return to its resting state and the muscle to relax (Rayment et al., 1993; Owen, Lamb & Stephenson, 1996; Xu & Van Remmen, 2021).

**Figure 2:**
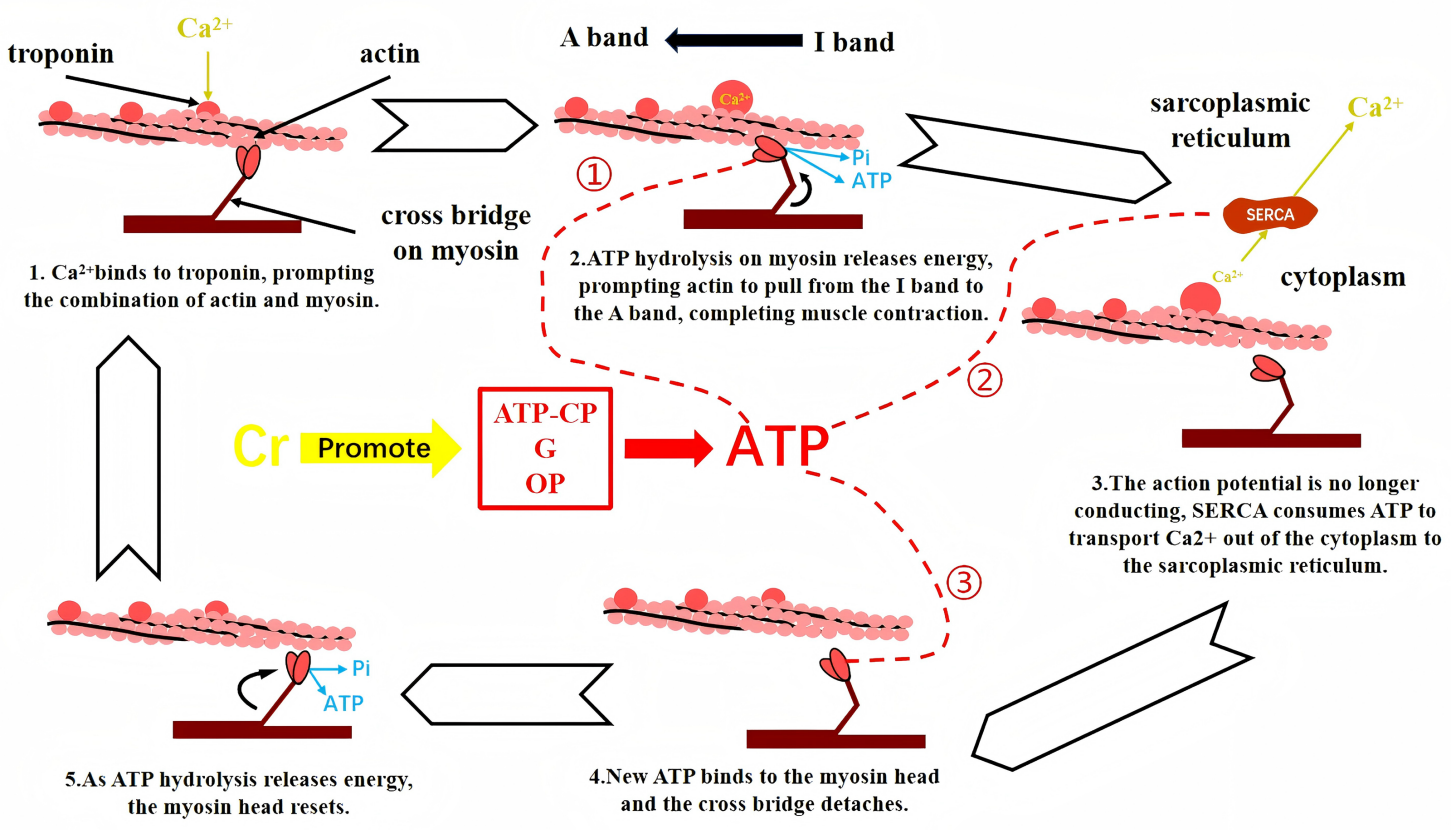
Cr supplementation and skeletal muscle myofilament gliding. Creatine supplementation drives three energy-producing processes, thereby promoting ATP production ①:The generated ATP first acts on myosin to provide energy for muscle fiber contraction ②:ATP also acts on SERCA to transport Ca 2+ a gainst the concentration gradient into the sarcoplasmic reticulum, preparmng for the next contraction ③:ATP finally acts on myosin to cause the myosin head to return to its initial position, preparing for the next contraction.

As stated above, ATP is essential for normal muscle contraction, but its reserves are limited and must be simultaneously broken down and synthesized to continuously meet the demands of muscular activity (Bonora et al., 2012). ATP synthesis originates from three pathways: the phosphagen system (ATP-CP), glycolysis (G), and oxidative phosphorylation (OP). In the body, most Cr is bound to form PCr. In addition to its own breakdown to provide energy for short-term explosive movements (ATP-CP pathway), PCr and Cr shuttle between mitochondria and the cytoplasm. This shuttling links ATP production sites (G and OP) with ATP utilization sites, thereby facilitating glycolysis and oxidative phosphorylation through the Cr shuttle mechanism (Kraft et al., 2000; Kay et al., 2000; Hespel et al., 2001; Wallimann, Tokarska-Schlattner & Schlattner, 2011). Given that Cr is a supplement that promotes energy regeneration, it is reasonable to conclude that Cr enhances users’ muscle strength by improving the body’s energy production efficiency (Fig. 2).

This study conducted a meta-analysis to systematically evaluate the effects of Cr supplementation on strength enhancement in the general population. It further analyzed variations in these effects across age-stratified groups and training backgrounds, while specifically investigating two critical issues in practical applications: the optimal dosage range for Cr supplementation and its compatible training intensity levels. Although previous studies have explored these issues, their conclusions remain limited in generalizability due to restricted sample sizes and high heterogeneity in results.

## Method

The methods of this meta-analysis and systematic review strictly followed the guidelines outlined in the Preferred Reporting Items for Systematic Reviews and Meta-Analyses (PRISMA) statement. This Meta-analysis has been registered on PROSPERO, with the registration number: CRD42024547697.

### Inclusion and exclusion criteria

Subjects included in the study were without restriction on age, gender, and training level. These subjects were divided into an experimental group and a control group using random grouping methods. Subjects in the experimental group received intervention with Cr powder, while subjects in the control group received intervention with a placebo highly similar to Cr powder in shape and taste. The intervention methods for both groups were identical, with both being administered by dissolving in warm water. During each intervention, the drinking containers were masked to prevent subjects from knowing which agent they were consuming. Additionally, in each included study, the two groups of subjects received identical interventions except for the pharmaceutical intervention protocols.

Included studies must provide direct and clear descriptions of baseline and post-supplementation strength test values. Muscle strength tests must be conducted using three common methods: leg press (LP), squat (SQ), and bench press (BP), with test units all in kilograms (Kg).

### Literature retrieval and data extraction

Literature retrieval was independently conducted by three authors, who searched keywords such as Cr, muscle strength, training, elderly, and sports performance in databases including PubMed, Cochrane Library, EMBASE, China National Knowledge Infrastructure (CNKI), and VIP Data. The search was not limited by specific years, languages, or institutions.

Data extraction was independently performed by two authors, who extracted key information from the finally included studies, such as the number of subjects in different groups, muscle strength test values before and after intervention, subject age, Cr dosage, training intensity, *etc*. After extraction, the data extracted by the two authors were compared with each other to check for consistency and ensure the accuracy of data extraction.

### Quality assessment of included studies

Quality assessment of included studies was conducted by three authors for risk of bias, using the risk of bias assessment tool in Review Manager 5.3 (Revman5.3) software provided by the Cochrane Collaboration. The evaluation indicators of the risk of bias scale in Revman5.3 software mainly include: (1) random sequence generation (selection bias); (2) allocation concealment (selection bias); (3) blinding of participants and researchers (performance bias); (4) blinding of outcome assessment (detection bias); (5) incomplete outcome data (attrition bias); (6) selective reporting (reporting bias); (7) other biases. Each indicator was assessed for risk as low risk, unclear risk, and high risk.

### Statistical analysis

#### RevMan 5.3 was employed for the main analysis

Meta-analysis was performed on the data from included studies using RevMan 5.3 software. The included studies were eligible for analysis of continuous variables. The effect size for continuous variables was expressed as the standardized mean difference (SMD) with its 95% confidence interval (CI). Heterogeneity among the included studies was assessed using the I^2^ test. When heterogeneity was low (*P* > 0.1, I^2^ < 50%), relevant data were pooled, and a fixed-effects model was used to calculate statistics; when heterogeneity was high (*P* ≤ 0.1, I^2^ ≥ 50%), potential sources of heterogeneity were explored, effect measures were not pooled without justification, and a random-effects model was applied for statistical calculations. Statistical significance was set at *P* < 0.05.

For each analysis, a meta-analysis was first conducted on the baseline values of the Cr group and the control group before Cr intervention to ensure no significant differences existed between the two groups at baseline. Subsequently, another meta-analysis was performed on the post-intervention values to observe whether significant differences emerged after the intervention. On the premise of no significant baseline differences, a significant difference (*P* ≤ 0.05) or highly significant difference (*P* < 0.01) in post-intervention data indicated a significant impact of the intervention on the tested. In subgroup analyses, the *P*-values of different subgroups post-intervention were compared to determine which subgroup exhibited a more pronounced intervention effect (lower *P*-values indicated greater significance). Forest plots generated by RevMan 5.3 were used as the visualization method to present the meta-analysis results.

#### Supplementary analysis: verifying result robustness by robust variance estimation

When conducting analyses with RevMan software, multiple muscle strength test indicators (BP, SQ, LP) from the same study may be included in the analyses (*e.g.*, overall analysis, age subgroup analysis, training level subgroup analysis, Cr dosage subgroup analysis). To address the issue of effect size dependence potentially caused by these multiple muscle strength test indicators within the same study, this study also employed the Robust Variance Estimation (RVE) method for sensitivity analysis, so as to verify the robustness of the main conclusions (Pustejovsky & Tipton, 2022). Specifically, in the overall analysis, we used the “Correlated and Hierarchical Effects (CHE)” working model, which takes into account both within-study heterogeneity (*ω*) and between-study heterogeneity (*τ*) to calculate effect sizes more robustly. In subgroup analyses, we also adopted the “Subgroup-Correlated Effects (SCE)” working model, which allows each different group in each distinct subgroup analysis to have an independent heterogeneity parameter *τ* (SCE), thereby reflecting independent between-study heterogeneity for each group of studies.

We referenced the assumption mentioned by Pustejovsky & Tipton that the sampling correlation coefficient (*ρ*) of within-study effect sizes is 0.6, and calculated small-sample corrected CR2-type robust standard errors, confidence intervals, and Wald tests using the clubSandwich package.All RVE analyses were completed using the metafor package and clubSandwich package in the R programming language.

## Result

### Literature search results

**Figure 3:**
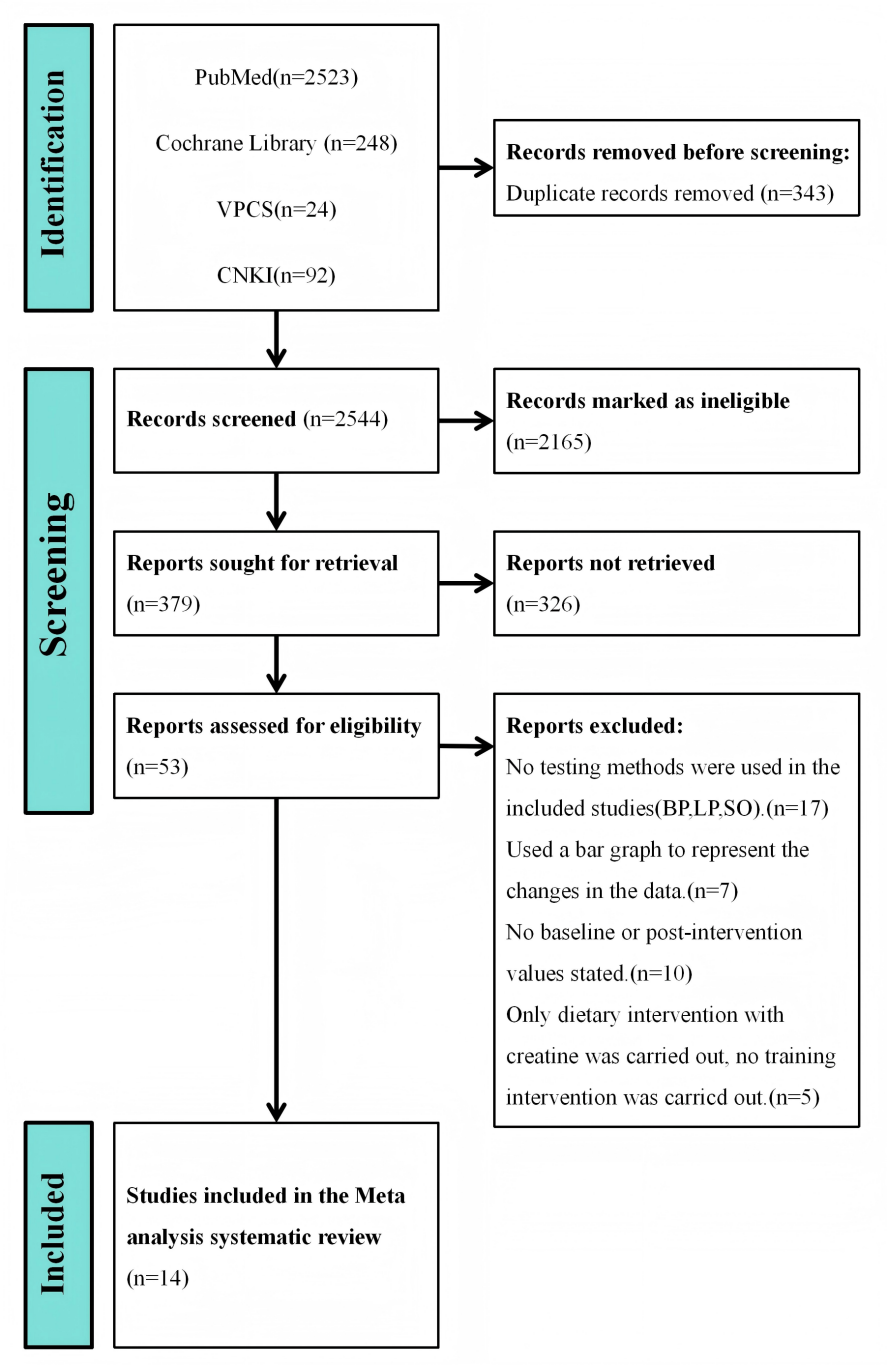
Study selection flow chart.

The initial literature search identified 2,887 articles related to the keywords. Of these, 343 duplicate studies were removed, 2,165 articles were excluded due to non-qualifying keywords in titles or abstracts, and an additional 326 articles were excluded as their study designs were irrelevant. This left 53 experimental studies investigating the effects of Cr supplementation on muscle strength gains. However, further analysis revealed the following exclusions: 17 studies involving soccer, rugby, or sprint athletes utilized sprint or countermovement jump tests as measurement outcomes, which did not meet this study’s inclusion criteria; seven studies presented muscle strength changes through bar graphs rather than numerical comparisons; 10 studies only described pre- to post-intervention changes in test scores without directly reporting baseline and post-intervention values; and five studies focusing on Cr supplementation in older adults involved dietary interventions only, with no exercise training. These studies were consequently excluded. Therefore, 14 articles were included in the meta-analysis (Vandenberghe et al., 1997; Stone et al., 1999; Syrotuik et al., 2001; Brose, Parise & Tarnopolsky, 2003; Law et al., 2009; Camic et al., 2014; Gualano et al., 2014; Candow et al., 2015; Candow et al., 2021; Wang et al., 2018; Kaviani, Abassi & Chilibeck, 2019; Bonilla et al., 2021; Samadi et al., 2022; Amiri & Sheikholeslami-Vatani, 2023) (Fig. 3).

### Raw data and intervention characteristics of included studies

The number of subjects in the included studies and the original muscle strength test data are shown in Table 1. The intervention characteristics of the included studies are presented in Table 2.

**Table 1:** Participant characteristics and muscle strength test values in included studies. The table summarizes the baseline values and post-intervention values of muscle strength tests from the included studies, all in kilograms (kg). Notably, some studies conducted multiple tests on the same participants, thus leading to multiple sets of test data being included in the meta-analysis.

**Study**	**Population**	**Test item**	**Muscle strength**			
			**Creatine group**		**Control group**	
			**Before**	**After**	**Before**	**After**
Amiri E (2023)	30 elderly individuals.(68.1 ± 7.2 years)	Bench press	32.15 ± 21.83	47 ± 31.53	19.62 ± 9.81	25.68 ± 14.40
Bonilla DA (2021)	15 trained young men.(26.6 ± 8.1 years)	Squat	110.62 ± 13.36	132.16 ± 17.72	113 ± 16.02	121.31 ± 19.87
Brose A (2003)	20 elderly individuals.(>65 years) 15 male,13 female	Leg press	M:88.076 ± 21.338 F:50.394 ± 12.712	M:110.776 ± 24.97 F:69.462 ± 24.516	M:75.364 ± 17.706 F:47.67 ± 11.804	M:104.874 ± 14.982 F:69.008 ± 13.62
Camic CL (2014)	50 untrained young men.(22.1 ± 2.5 years)	Bench press	88.5 ± 4.0	92.8 ± 3.7	87.0 ± 4.1	89.2 ± 4.1
Candow DG (2015)	27 elderly individuals.(>50 years) 10 males,17 females	Bench press	50.0 ± 26.2	65.2 ± 33.6	49.3 ± 20.2	51.2 ± 16.4
Candow DG (2020)	46 elderly male individuals.(49–69 years)	Squat	111 ± 25	180 ± 27	105 ± 24	181 ± 28
		Bench press	97 ± 15	108 ± 13	92 ± 15	114 ± 14
Gualano B (2014)	30 elderly women.(>60 years)	Bench press	33.9 ± 5.6	36.5 ± 7.1	31.2 ± 7.9	33.0 ± 4.9
		Leg press	83.8 ± 19.4	97.7 ± 21.7	75.5 ± 14.2	85.5 ± 13.9
Kaviani M (2019)	18 untrained male youths.(22 ± 3 years)	Bench press	73 ± 9	85 ± 2	72 ± 4	79 ± 6
		Leg press	115 ± 21	162 ± 16	114 ± 24	147 ± 12
Law YL (2009)	17 trained male youths.(23.11 ± 3.56 years)	Bench press	73.75 ± 9.91	77.19 ± 10.81	68.75 ± 15.70	74.38 ± 17.26
		Squat	99.69 ± 13.26	110.94 ± 15.00	108.44 ± 19.86	109.69 ± 15.00
Samadi M (2022)	20 trained young men.(21 ± 1.5 years)	Leg press	202 ± 16.4	205 ± 17.71	201.5 ± 20.4	204.16 ± 19.47
Stone MH (1999)	20 trained young men.(18.4 ± 0.5 years)	Squat	149.7 ± 9.0	167 ± 9.5	149.8 ± 11.1	162.2 ± 10.3
		Bench press	124.5 ± 6.3	136.9 ± 6.6	129.1 ± 5.6	134.1 ± 5.7
Syrotuik DG (2001)	23 trained youths.(23 years) 12 males, 11 females	Bench press	46.8 ± 14.5	49.1 ± 15.5	48.2 ± 19.5	50.9 ± 20.9
Vandenberghe K (1997)	19 untrained female youths.(19-22 years)	Squat	25.878 ± 1.362	37.682 ± 0.27	25.878 ± 1.816	32.234 ± 2.724
		Bench press	21.338 ± 0.908	30.872 ± 1.816	21.792 ± 1.362	29.964 ± 1.816
Wang CC (2018)	30 pre-trained male youths.(20 ± 2 years)	Squat	133.67 ± 14.07	178.33 ± 16.86	131.67 ± 15.77	165.66 ± 14.62

**Notes.**

Vandenberghe et al. (1997); Stone et al. (1999); Syrotuik et al. (2001); Brose, Parise & Tarnopolsky (2003); Law et al. (2009); Camic et al. (2014); Gualano et al. (2014); Candow et al. (2015); Candow et al. (2021); Wang et al. (2018); Kaviani, Abassi & Chilibeck (2019); Bonilla et al. (2021); Samadi et al. (2022); Amiri & Sheikholeslami-Vatani (2023).

**Table 2:** Intervention characteristics of included studies. The table summarizes the intervention characteristics of the included studies, including the creatine intervention dosage (notably, studies that did not specify the dosage are denoted by “/” in the table) and training intervention features (training intensity explicitly mentioned in the studies is presented in bold font).

**STUDY**	**Creatine dosage**	**Training intervention characteristics**
Amiri E (2023)	\	A 10-week program consisting of three resistance training sessions per week
Bonilla DA (2021)	7.56 g/d	An 8-week program consisting of three high-intensity resistance training sessions per week (90%1RM)
Brose A (2003)	\	A 14-week program conssting of three progressive resistance training sessions per week (50%–80%1RM)
Camic CL (2014)	2.5 g/d	A 4-week period of daily physical activity
Candow DG (2015)	\	A 32-week program of self-selected intensity training
Candow DG (2020)	\	A 12-month program consisting of three whole-body resistance training sessions per week
Gualano B (2014)	\	A 24-week program consisting of two resistance training sessions per week
Kaviani M (2019)	5.15 g/d	An 8-week program consisting of three resistance training sessions per week (75%1RM)
Law YL (2009)	20 g/d	A program of 8 weeks with 3 resistance training sessions per week (60%1RM)
Samadi M (2022)	23.55 g/d	A 4-week military training program
Stone MH (1999)	19.5 g/d	A 7-week high-intensity football-specific training program
Syrotuik DG (2001)	4.89 g/d	A 9-week program consisting of two resistance training sessions per week and four rowing-specific training sessions per week
Vandenberghe K (1997)	5.8 g/d	A 10-week program consisting of three resistance training sessions per week (70%1RM)
Wang CC (2018)	5.18 g/d	A 4-week program consisting of three high-intensity training sessions per week (80%1RM)

**Notes.**

Vandenberghe et al. (1997); Stone et al. (1999); Syrotuik et al. (2001); Brose, Parise & Tarnopolsky (2003); Law et al. (2009); Camic et al. (2014); Gualano et al. (2014); Candow et al. (2015); Candow et al. (2021); Wang et al. (2018); Kaviani, Abassi & Chilibeck (2019); Bonilla et al. (2021); Samadi et al. (2022); Amiri & Sheikholeslami-Vatani (2023).

### Risk of bias assessment results of included studies

**Figure 4:**
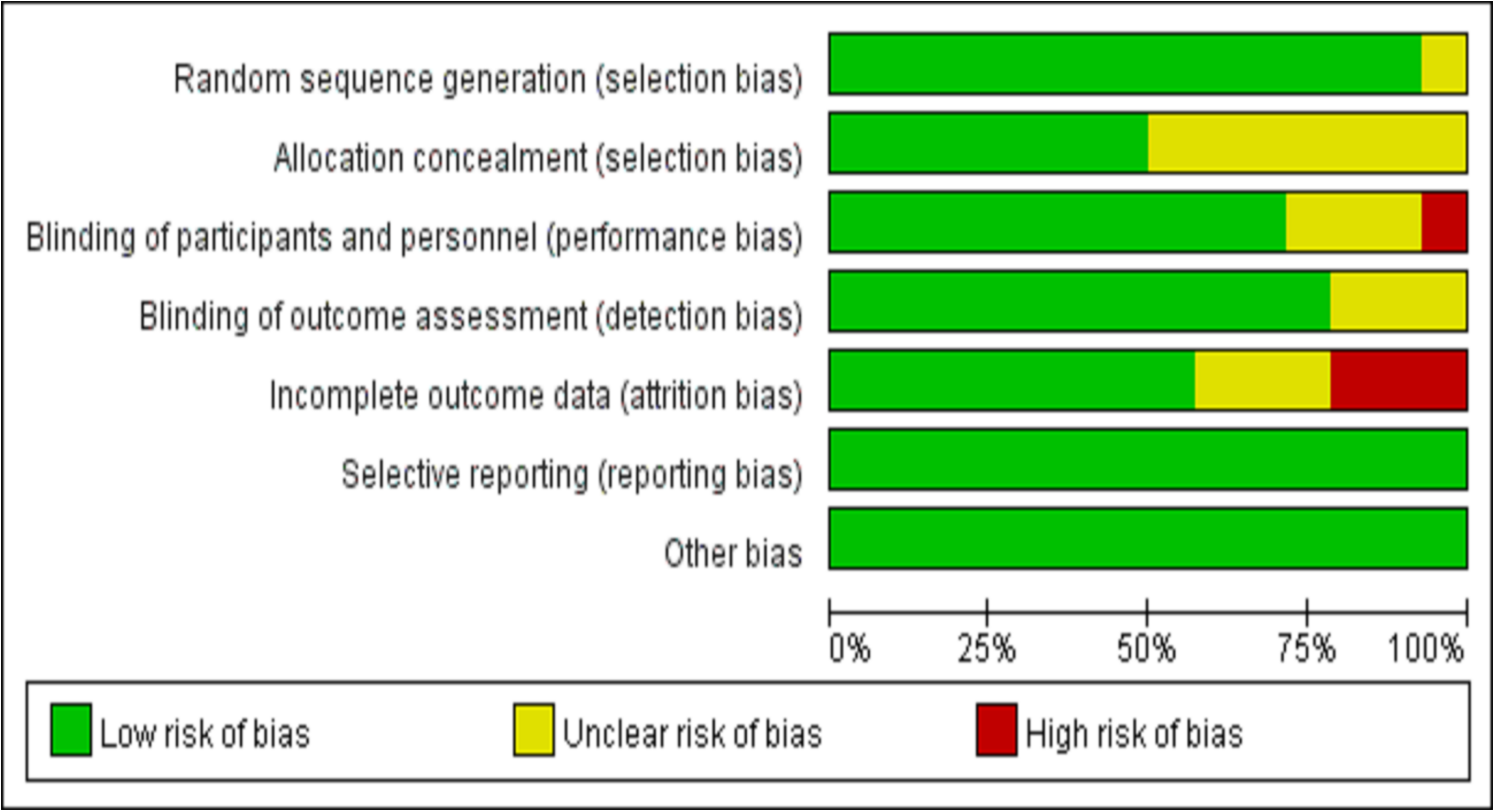
Quality evaluation of the included studies. The risk of bias for all included studies was summarized using the Cochrane Collaboration’s risk of bias tool in Revman 5.3. The figure displays the proportions of studies with low (green), unclear (yellow), and high (red) risk of bias in each assessment domain.

**Figure 5:**
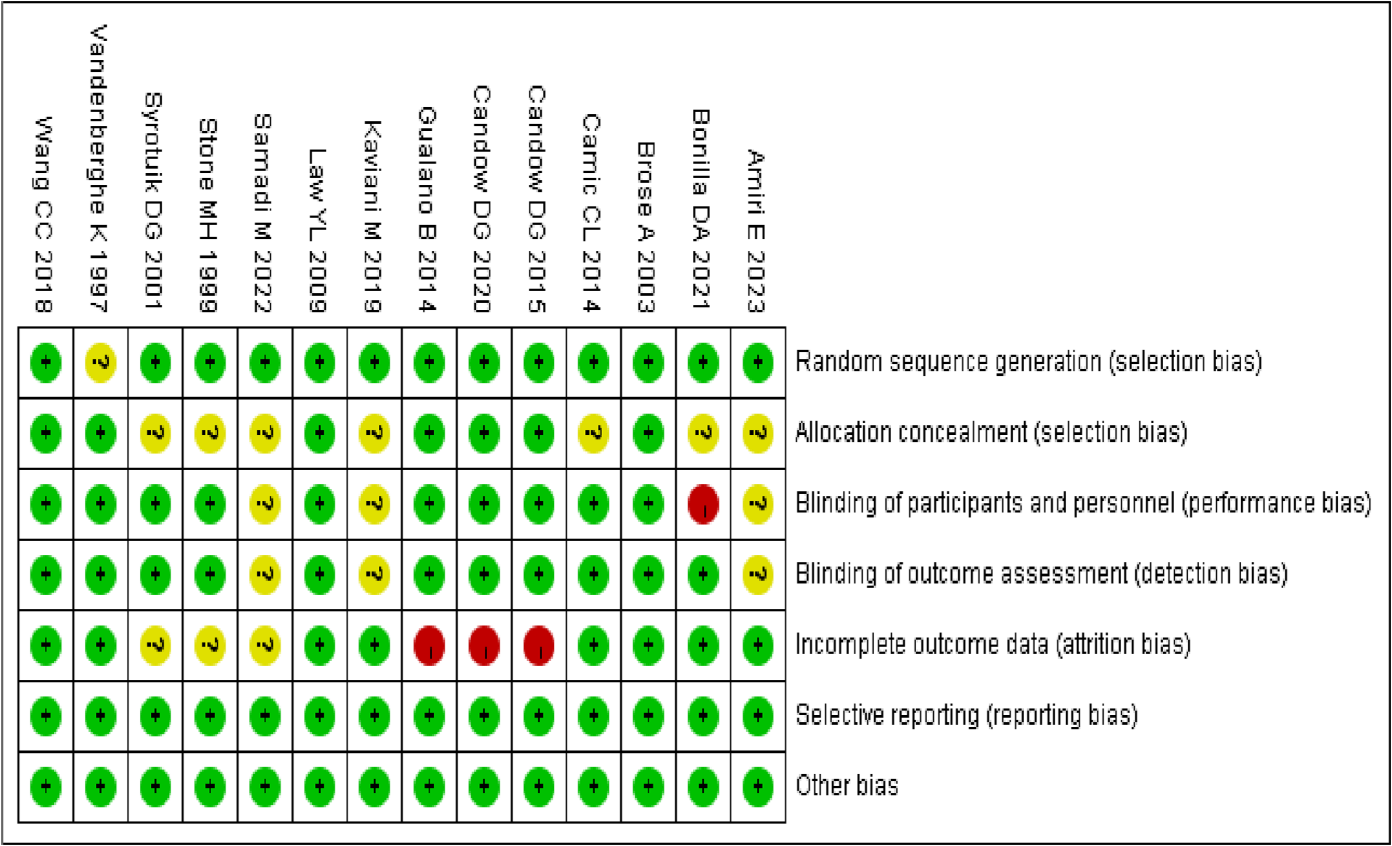
Evaluation summary of the included studies. Each study was assessed across seven domains: random sequence generation, allocation concealment, blinding of participants and personnel, blinding of outcome assessment, incomplete outcome data, selective reporting, and other bias sources. Green (+) indicates low risk of bias, yellow (?) indicates unclear risk, and red (-) indicates high risk (Vandenberghe et al., 1997; Stone et al., 1999; Syrotuik et al., 2001; Brose, Parise & Tarnopolsky, 2003; Law et al., 2009; Camic et al., 2014; Gualano et al., 2014; Candow et al., 2015; Candow et al., 2021; Wang et al., 2018; Kaviani, Abassi & Chilibeck, 2019; Bonilla et al., 2021; Samadi et al., 2022; Amiri & Sheikholeslami-Vatani, 2023).

Among the 14 included studies, 13 described specific methods for random sequence generation. Regarding allocation concealment, it was implemented in seven studies. For blinding, double-blind procedures were used in 10 studies, single-blind in one study, and three studies did not report blinding details. Three studies reported participant dropout, while no studies indicated reporting bias; however, the risk of other potential biases remained unclear (Figs. 4 and 5).

### Meta-analysis results

All 14 screened studies were included in the analysis. As some studies involved the same participants undergoing multiple strength tests, the pooled dataset comprised experimental data from 523 participants: 259 in the Cr group and 264 in the control group (Vandenberghe et al., 1997; Stone et al., 1999; Law et al., 2009; Gualano et al., 2014; Kaviani, Abassi & Chilibeck, 2019; Candow et al., 2021). The majority of participants were male, with ages ranging from 19 to 69 years.

#### Overall analysis results

**Figure 6:**
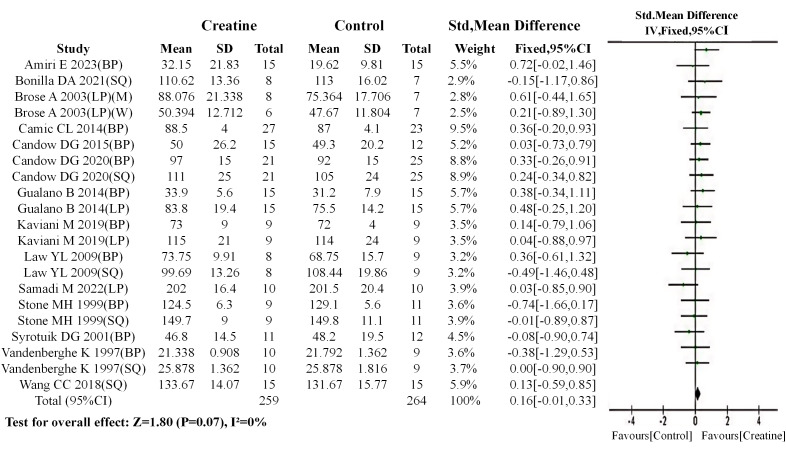
Meta-analysis dendrogram of overall baseline values. The forest plot displays the standardized mean differences (SMD) and 95% confidence intervals (CI) across included studies, along with the statistical significance (*P*-values) of between-group differences. Results showed no significant difference in baseline values between the creatine and control groups (*P* = 0.07, SMD = 0.16, 95% CI [−0.01–0.33]), and the overall data exhibited no heterogeneity (I^2^ = 0%) (Vandenberghe et al., 1997; Stone et al., 1999; Syrotuik et al., 2001; Brose, Parise & Tarnopolsky, 2003; Law et al., 2009; Camic et al., 2014; Gualano et al., 2014; Candow et al., 2015; Candow et al., 2021; Wang et al., 2018; Kaviani, Abassi & Chilibeck, 2019; Bonilla et al., 2021; Samadi et al., 2022; Amiri & Sheikholeslami-Vatani, 2023).

A meta-analysis was initially conducted to compare baseline values between the Cr and control groups prior to intervention. The results demonstrated no significant between Cr group and control group in the 14 included studies (SMD = 0.16, 95% CI [−0.01–0.33]; I^2^ = 0%, *P* = 0.07) (Fig. 6). When baseline equivalence (*P* > 0.05) is observed with sufficient sample size, the baseline data can be considered homogeneous. This establishes a valid foundation for comparing post-intervention effects between the two groups.

**Figure 7:**
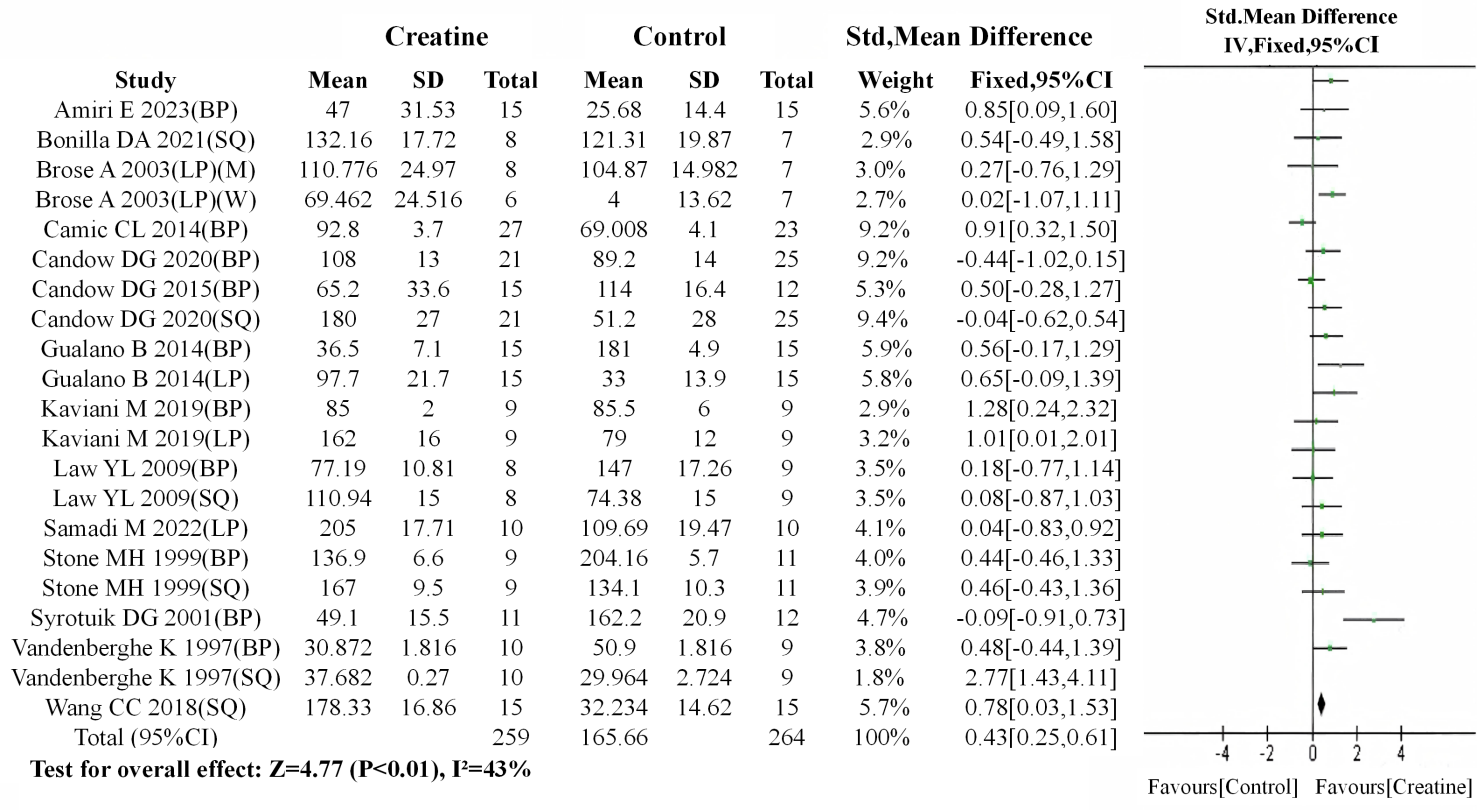
Meta-analysis dendrogram after overall intervention. After intervention, there was a highly significant difference in muscle strength test values between the creatine and control groups (*P* < 0.01, SMD = 0.43, 95% CI [0.25–0.61]). The overall data showed moderate heterogeneity (I^2^ = 43%), with heterogeneity predominantly attributed to the creatine group (Vandenberghe et al., 1997; Stone et al., 1999; Syrotuik et al., 2001; Brose, Parise & Tarnopolsky, 2003; Law et al., 2009; Camic et al., 2014; Gualano et al., 2014; Candow et al., 2015; Candow et al., 2021; Wang et al., 2018; Kaviani, Abassi & Chilibeck, 2019; Bonilla et al., 2021; Samadi et al., 2022; Amiri & Sheikholeslami-Vatani, 2023).

Following the baseline meta-analysis, a subsequent meta-analysis of post-intervention data between the Cr and control groups revealed a highly significant difference favoring the Cr group, with measurable heterogeneity skewed toward Cr supplementation benefits (SMD = 0.43, 95% CI: 0.25 to 0.61; I^2^ = 43%, *P* < 0.01) (Fig. 7).

Since multiple test values from each of some studies were included in the analysis, the CHE model in the RVE sensitivity analysis method was employed for further verification to correct for the dependence of effect sizes. The results showed that Cr intervention still significantly improved muscle strength in the general population (SMD = 0.451, 95% CI [0.032–0.871], *p* = 0.02) (Table 3).

**Table 3:** Overall meta-analysis with RVE.

**Term**	**Estimate (SMD)**	**Std.Error**	**95% CI lower**	**95% CI upper**	***P*-value**
intrct	0.451	0.195	0.032	0.871	0.02
*τ*	0.359				
*ω*	0.581				

Synthesizing the above analyses reveals that: among the same group of participants, there was no significant difference in baseline values between the two groups; however, after receiving different interventions respectively, the two groups exhibited a significant difference in post-intervention test values, and the heterogeneity was skewed toward the Cr group. This indicates that, compared with the placebo-supplemented control group, the Cr-supplemented Cr group had a more significant effect on participants’ muscle strength gain under the condition of the same training intervention.

#### Subgroup analysis results for middle-aged/elderly and young adults

Age information of the study participants was collected, and according to the World Health Organization (WHO) standards with a cutoff of 24 years (World Health Organization, 1987), the included participants were divided into the middle-aged and elderly (49–69 years) group (95 in the Cr group and 96 in the control group) (Brose, Parise & Tarnopolsky, 2003; Gualano et al., 2014; Candow et al., 2015, Candow et al., 2021; Amiri & Sheikholeslami-Vatani, 2023) and the young (19–23 years) group (88 in the Cr group and 93 in the control group) (Vandenberghe et al., 1997; Stone et al., 1999; Syrotuik et al., 2001; Law et al., 2009; Wang et al., 2018; Bonilla et al., 2021; Samadi et al., 2022).

**Figure 8:**
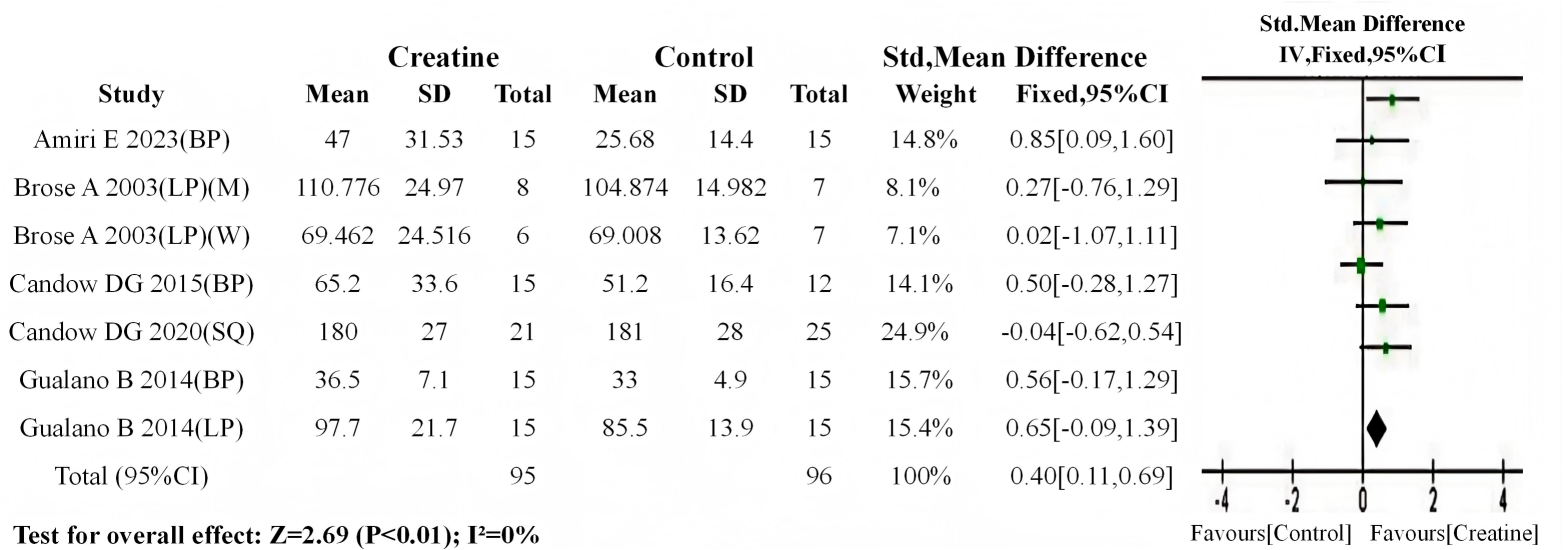
Meta-analysis dendrogram of middle-aged and elderly people after intervention. In the middle-aged and elderly subgroup, post-intervention muscle strength test values differed significantly between creatine and control groups (*P* < 0.01, SMD = 0.40, 95% CI [0.11–0.69]), with no heterogeneity observed (I^2^ = 0%) (Amiri & Sheikholeslami-Vatani, 2023; Brose, Parise & Tarnopolsky, 2003; Candow et al., 2015; Candow et al., 2021; Gualano et al., 2014).

**Figure 9:**
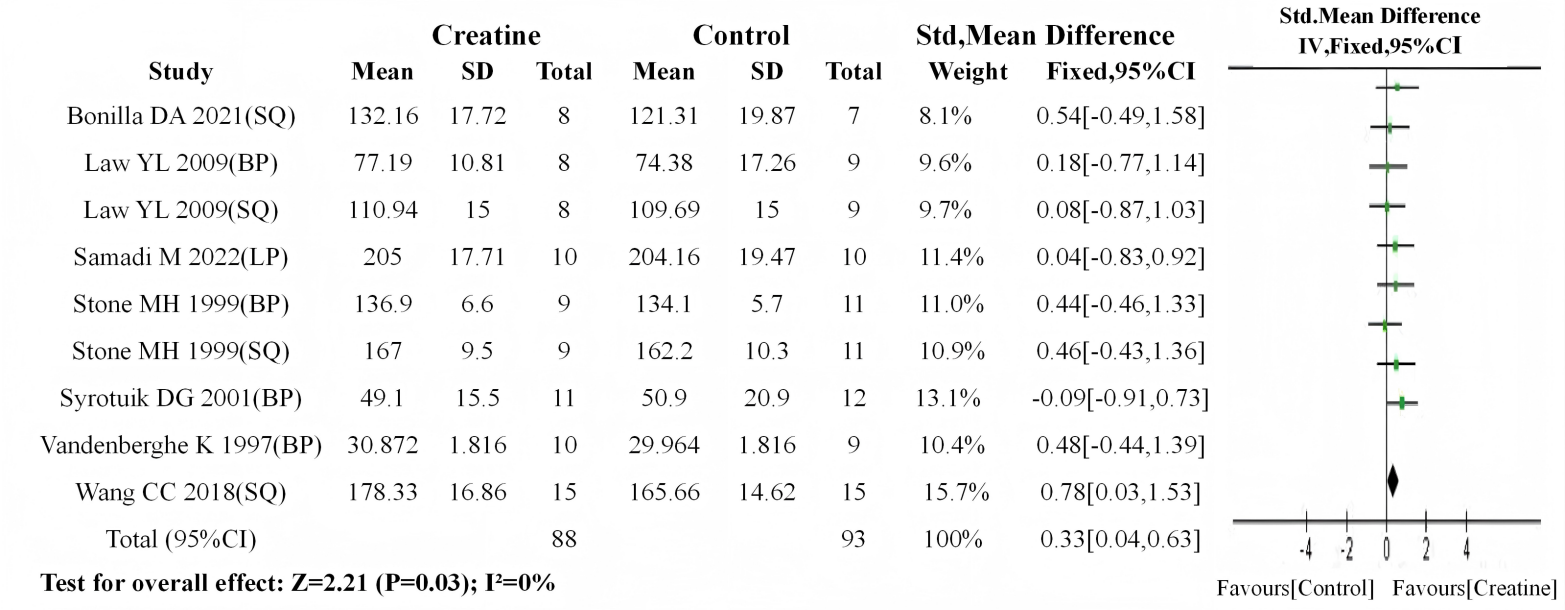
Meta-analysis dendrogram of young people group after intervention. In the young adult subgroup, post-intervention values showed a significant difference (*P* = 0.03, SMD = 0.33, 95% CI [0.04–0.63]), with no heterogeneity (I^2^ = 0%) (Vandenberghe et al., 1997; Stone et al., 1999; Syrotuik et al., 2001; Law et al., 2009; Wang et al., 2018; Bonilla et al., 2021; Samadi et al., 2022).

Following the analytical protocol used in the overall analysis, baseline meta-analyses were first conducted for both subgroups. Results indicated no significant baseline differences in either subgroup (older adults: *P* = 0.06; younger adults: *P* = 0.40). Subsequent meta-analyses of post-intervention data revealed: (1) a highly significant between-group difference in older adults (SMD = 0.40, 95% CI [0.11–0.69]; I^2^ = 0%, *P* < 0.01) (Fig. 8); (2) a statistically significant difference in younger adults (SMD = 0.33, 95% CI [0.04–0.63]; I^2^ = 0%, *P* = 0.03) (Fig. 9).

Similarly, since multiple test values from some studies were included in the analysis, the CHE and SCE models in the RVE sensitivity analysis method were employed for robustness analysis of effects. However, there were differences in the results compared with the conventional meta-analysis mentioned above: Under the CHE model, the estimated effect size of the young group (SMD = 0.601) was higher than that of the middle-aged and elderly group (SMD = 0.107), and the between-group difference did not reach statistical significance (*p* = 0.085). The SCE model showed a similar trend (young group: SMD = 0.592; middle-aged and elderly group: SMD = 0.100; *p* = 0.097) (Table 4).

**Table 4:** Subgroup meta-analysis for middle-aged/elderly and young adults with RVE.

			**Correlated hierarchical effects (CHE)**	**Sub-group correlated effects (SCE)**	
**Age group**	**Studies**	**Effect sizes**	**Est (SMD).[SE]**	**Est (SMD).[SE]**	**tau (SCE)**
Youth	7	9	0.601 [0.190]	0.592 [0.192]	0.238
Old	6	7	0.107 [0.175]	0.100 [0.190]	0.244
*τ*			0.000		
*ω*			0.341		
Wald test *p*-value			0.085	0.097	

#### Subgroup analysis results of trained and untrained populations

According to the descriptive information of the participants included in the study, using whether they had received professional training (training duration longer than three months, with professional coaches, venues, and facilities, *etc*.) as the criterion, the participants were divided into the trained group (78 in the Cr group and 84 in the control group) (Stone et al., 1999; Syrotuik et al., 2001; Law et al., 2009; Wang et al., 2018; Bonilla et al., 2021) and the untrained group (65 in the Cr group and 59 in the control group) (Vandenberghe et al., 1997; Camic et al., 2014; Kaviani, Abassi & Chilibeck, 2019).

**Figure 10:**
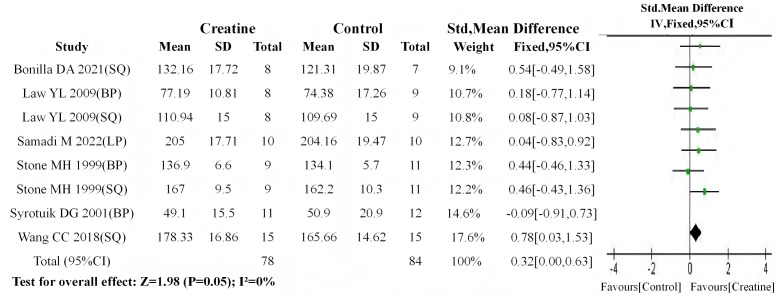
Meta-analysis dendrogram of the trained group after intervention. In the trained subgroup, post-intervention values differed significantly (*P* = 0.05, SMD = 0.32, 95% CI [0.00–0.63]), with no heterogeneity (I^2^ = 0%) (Stone et al., 1999; Syrotuik et al., 2001; Law et al., 2009; Wang et al., 2018; Bonilla et al., 2021; Samadi et al., 2022).

**Figure 11:**
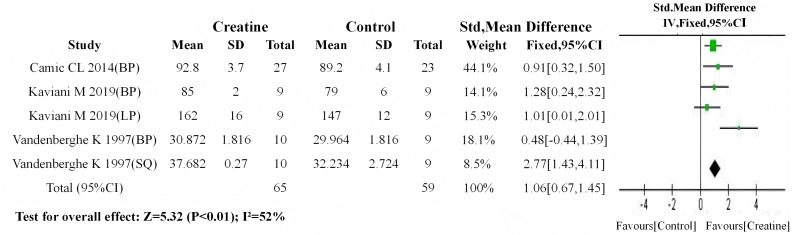
Meta-analysis dendrogram of the untrained group after intervention. In the untrained subgroup, post-intervention values showed a highly significant difference (*P* < 0.01, SMD = 1.06, 95% CI [0.67–1.45]), accompanied by moderate heterogeneity (I^2^ = 52%) (Vandenberghe et al., 1997; Kaviani, Abassi & Chilibeck, 2019; Camic et al., 2014).

Consistent with the aforementioned methodology, initial meta-analyses of baseline values demonstrated no significant between-group differences in either subgroup (trained: *P* = 0.54; untrained: *P* = 0.53). Subsequent post-intervention meta-analyses revealed: (1) a statistically significant improvement in muscle strength for the trained subgroup following Cr supplementation (SMD = 0.32, 95% CI [0.00–0.63]; I^2^ = 0%, *P* = 0.05) (Fig. 10); (2) a highly significant enhancement in the untrained subgroup (SMD = 1.06, 95% CI [0.67–1.45]; I^2^ = 52%, *P* < 0.01) (Fig. 11).

Results from the further robust RVE sensitivity analysis were consistent with those from the conventional meta-analysis mentioned above. Under the CHE model, the estimated effect size of the untrained group (SMD = 1.216) was higher than that of the trained group (SMD = 0.562). The SCE model showed similar results: the estimated effect size of the untrained group was larger than that of the trained group (untrained: SMD = 1.158; trained: SMD = 0.530). However, whether under the CHE model or the SCE model, the between-group differences in effect sizes were not significant (CHE-p = 0.266; SCE-p = 0.297) (Table 5).

**Table 5:** Subgroup meta-analysis for trained and untrained populations with RVE.

			**Correlated hierarchical effects**	**Sub-group correlated effects**	
**Train group**	**Studies**	**Effect sizes**	**Est.[SMD] (CHE)**	**Est.[SMD] (SCE)**	**tau (SCE)**
trained	6	8	0.562 [0.211]	0.530 [0.209]	0.260
untrained	3	5	1.216 [0.466]	1.158 [0.445]	0.659
*τ*			0.000		
*ω*			0.688		
Wald test *p*-value			0.266	0.297	

#### Subgroup analysis results of high and low doses of cr supplementation

Studies with clear records of Cr supplementation dosage were screened from the included studies. A supplementation dosage greater than 15 g/d was defined as high dose, and less than 10 g/d was defined as low dose (Ainsley Dean et al., 2017). Accordingly, the included studies were divided into a high-dose group (19.5–23.55 g/d) and a low-dose group (2.5–7.56 g/d). Among them, the high-dose group included three studies (Stone et al., 1999; Law et al., 2009; Samadi et al., 2022) (44 in the Cr group, 50 in the control group), and the low-dose group included 6 studies (Vandenberghe et al., 1997; Syrotuik et al., 2001; Camic et al., 2014; Wang et al., 2018; Kaviani, Abassi & Chilibeck, 2019; Bonilla et al., 2021) (80 in the Cr group, 75 in the control group).

**Figure 12:**
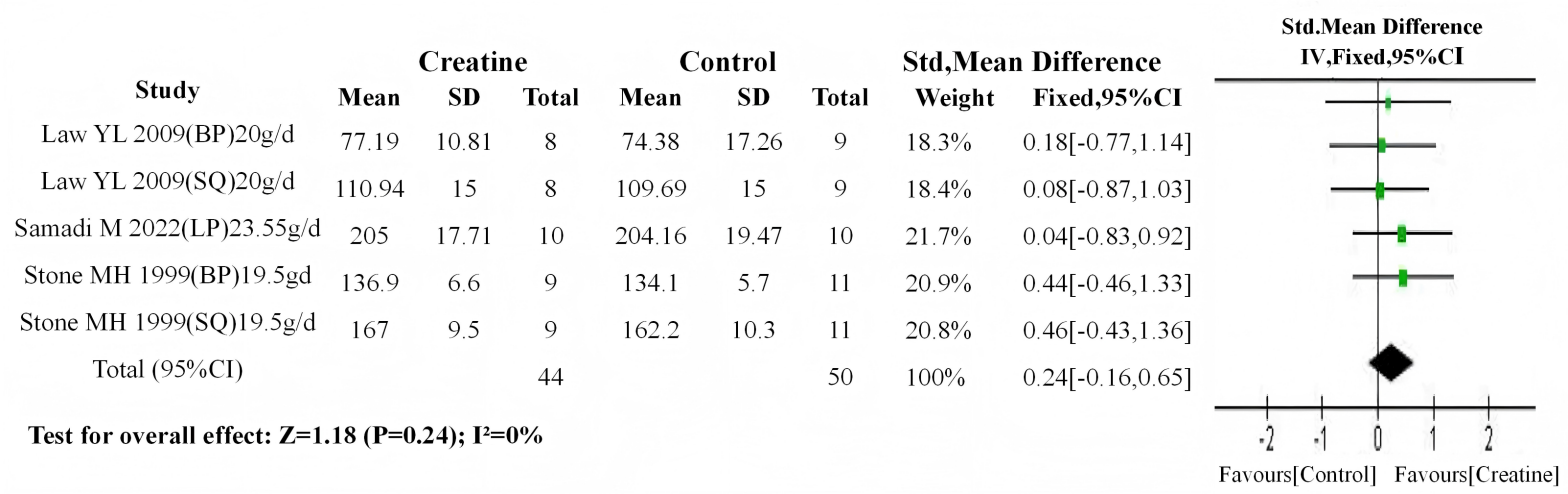
Meta-analysis dendrogram of high-dose Cr supplementation group. In the high-dose creatine subgroup, post-intervention values showed no significant difference (*P* = 0.24, SMD = 0.24, 95% CI [−0.16–0.65]), with no heterogeneity (I^2^ = 0%) (Stone et al., 1999; Law et al., 2009; Samadi et al., 2022).

**Figure 13:**
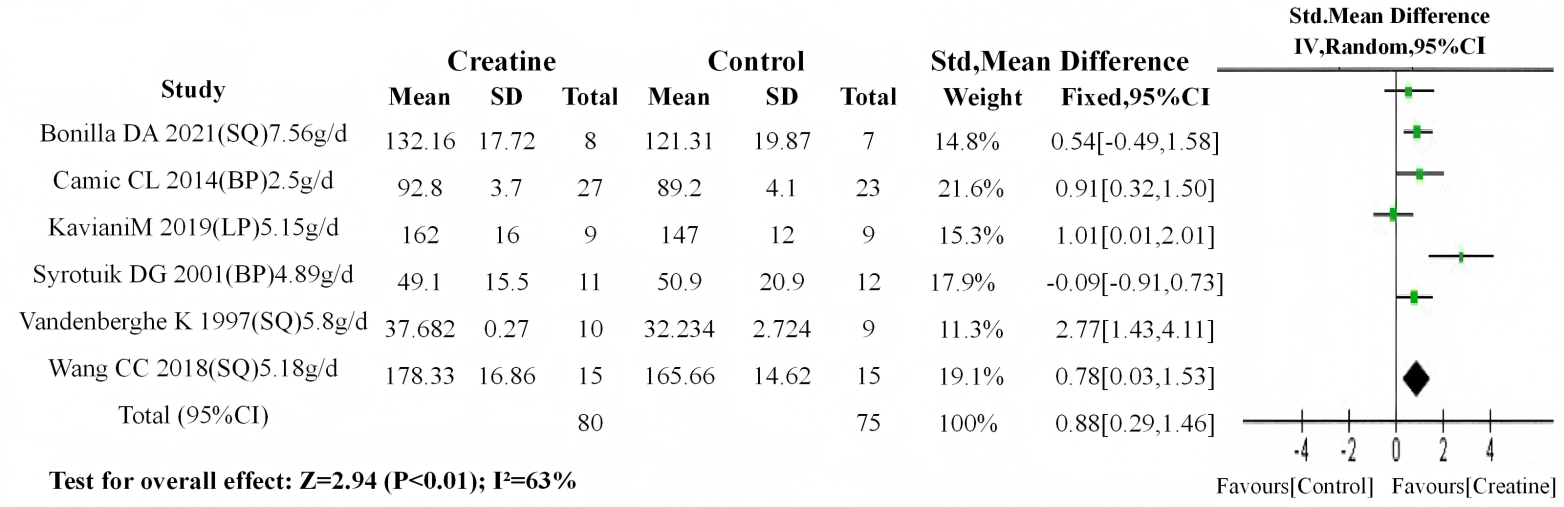
Meta-analysis dendrogram of low-dose Cr supplementation group. In the low-dose creatine subgroup, post-intervention values differed highly significantly (*P* < 0.01, SMD = 0.88, 95% CI [0.29–1.46]), with moderate heterogeneity (I^2^ = 63%) (Vandenberghe et al., 1997; Syrotuik et al., 2001; Camic et al., 2014; Wang et al., 2018; Kaviani, Abassi & Chilibeck, 2019; Bonilla et al., 2021; Amiri & Sheikholeslami-Vatani, 2023).

Baseline meta-analyses showed no significant between-group differences in either dosage subgroup (high-dose: *P* = 0.4; low-dose: *P* = 0.5). Subsequent post-intervention analyses demonstrated: (1) non-significant differences in the high-dose subgroup (SMD = 0.24, 95% CI [−0.16–0.65]; I^2^ = 0%, *P* = 0.24) (Fig. 12); (2) highly significant differences favoring Cr supplementation in the low-dose subgroup (SMD = 0.88, 95% CI [0.29–1.46]; I^2^ = 63%, *P* < 0.01) (Fig. 13).

Consistent with the results of the conventional meta-analysis method, the results of the RVE sensitivity analysis showed that whether under the CHE model (low dose: SMD = 0.988; high dose: SMD = 0.528) or the SCE model (low dose: SMD = 1.019; high dose: SMD = 0.478), the pooled effect size of the low-dose group was larger than that of the high-dose group. Moreover, whether under the CHE model or the SCE model, the difference in effect sizes between the two groups was not significant (CHE-p = 0.403; SCE-p = 0.330) (Table 6).

**Table 6:** Subgroup meta-analysis for high and low doses of cr supplementation with RVE.

			**Correlated hierarchical effects**	**Sub-group correlated effects**	
**Dose group**	**Studies**	**Effect sizes**	**Est[SMD] (CHE)**	**Est.[SMD] (SCE)**	**tau (SCE)**
High dose	3	5	0.528 [0.331]	0.478 [0.328]	0.355
low dose	6	6	0.988 [0.373]	1.019 [0.396]	0.864
*τ*			0.248		
*ω*			0.661		
Wald test *p*-value			0.403	0.330	

#### Subgroup analysis results of training intensity matching with Cr supplementation

Cr supplementation is predominantly combined with exercise training to augment muscle strength gains; however, the exercise training intensity implemented across studies varies substantially. This study hypothesizes that differential training intensities may modulate the ergogenic effects of Cr on strength enhancement. To address this hypothesis, subgroup analyses stratified by training intensity were conducted.

From all included studies, those specifying training intensity (standardized as percentage of one-repetition maximum, %1RM) were selected. Using 75%1RM as the cutoff (Haniel, 2025), studies were stratified into two subgroups: moderate-to-high intensity intervention (training intensity > 75%1RM; Cr group *n* = 32, control *n* = 34; three studies) (Stone et al., 1999; Wang et al., 2018; Bonilla et al., 2021) and moderate-to-low intensity intervention (training intensity <75%1RM; Cr group *n* = 27, control *n* = 27; three studies) (Vandenberghe et al., 1997; Law et al., 2009; Kaviani, Abassi & Chilibeck, 2019e).

**Figure 14:**
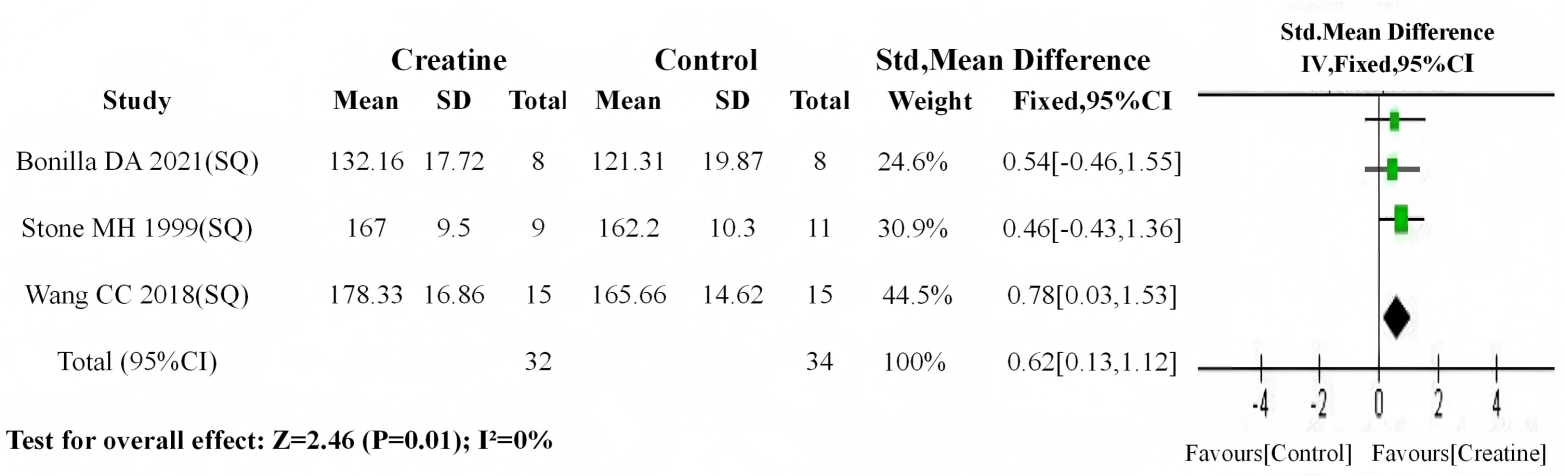
Meta analysis dendrogram of medium and high training intensity group. In the moderate-high training intensity subgroup, post-intervention values showed a significant difference (*P* = 0.01, SMD = 0.62, 95% CI [0.13–1.12]), with no heterogeneity (I^2^ = 0%) %) (Stone et al., 1999; Wang et al., 2018; Bonilla et al., 2021).

**Figure 15:**
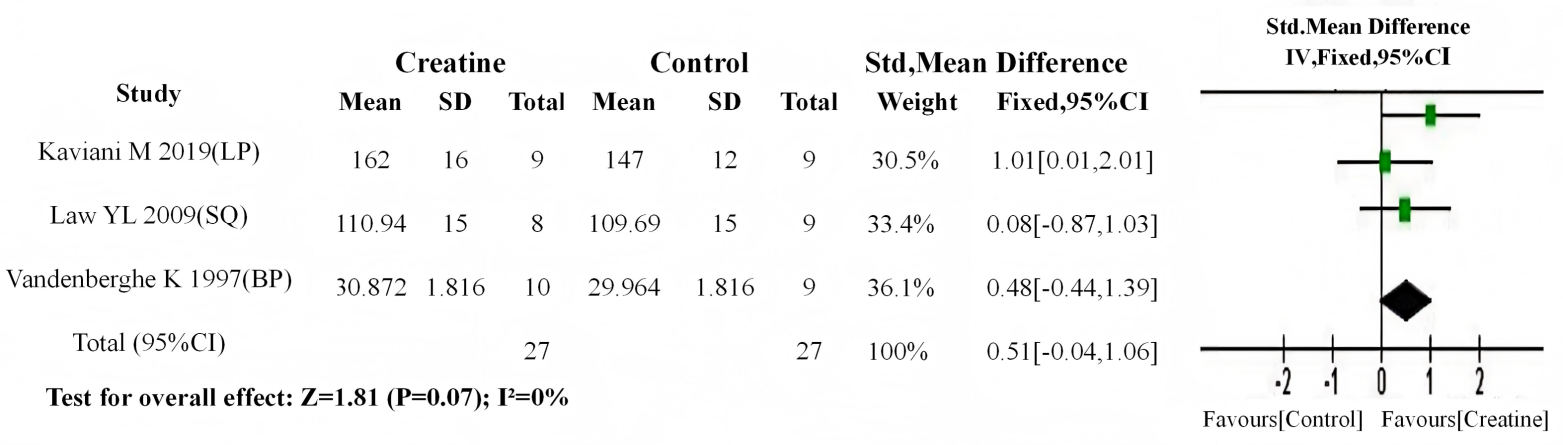
Meta analysis dendrogram of medium and low training intensity groups. In the moderate-low training intensity subgroup, post-intervention values showed no significant difference (*P* = 0.07, SMD = 0.51, 95% CI [−0.04–1.06]), with no heterogeneity (I^2^ = 0%) (Vandenberghe et al., 1997; Law et al., 2009; Kaviani, Abassi & Chilibeck, 2019).

Baseline meta-analyses revealed no significant differences in either subgroup (moderate-to-high: *P* = 0.94; moderate-to-low: *P* = 0.33). Subsequent post-intervention analyses demonstrated: (1) significant between-group differences in the moderate-to-high intensity subgroup (SMD = 0.62, 95% CI [0.13–1.12]; I^2^ = 0%, *P* = 0.01) (Fig. 14); (2) non-significant differences in the moderate-to-low intensity subgroup (SMD = 0.51, 95% CI [−0.04–1.06]; I^2^ = 0%, *P* = 0.07) (Fig. 15). These findings suggest that Cr supplementation paired with moderate-to-high intensity training produces more substantial muscle strength gains compared to moderate-to-low intensity regimens.

## Discussion

### Overall analysis

Studies included in this meta-analysis covered most of the Cr supplementation and training protocols, with participants’ ages also showing a wide distribution. Furthermore, validation *via* the CHE model in the RVE sensitivity analysis method further strengthened the validity of the overall meta-analysis results. Thus, it can be considered that the results of this meta-analysis have strong generalizability, that is, exogenous Cr supplements have a generally significant effect on muscle strength gain.

However, not all included studies demonstrated significant strength-enhancing effects of Cr supplementation: Candow et al. (2021) reported inferior strength gains in the Cr group compared to placebo controls. Notably, this trial exclusively enrolled older adults (49–69 years) and implemented high-intensity progressive resistance training (70–80% 1RM) in both Cr and placebo groups, characteristics seemingly incongruent with our findings. Nevertheless, this does not invalidate our analytical conclusions, as another study by Candow et al. (2015)—employing comparable intervention protocols (targeting older adults)—demonstrated statistically significant strength improvements in the Cr group *versus* controls. Comparative analysis revealed that Candow et al. (2021) utilized an exceptionally prolonged 12-month intervention period. Such extended supplementation likely induced a plateau in intramuscular Cr pool saturation within weeks, thereby diminishing the ergogenic potential of sustained exogenous Cr administration (Antonio et al., 2025). In contrast, longitudinal progressive resistance training adapts loading parameters according to strength progression; for older populations with baseline strength deficits, this adaptive protocol prevents pronounced performance plateaus over extended periods (Bloch-Ibenfeldt et al., 2024). Consequently, the discordant outcomes in Candow et al. (2021) are more plausibly attributable to Cr metabolic homeostasis shifts induced by ultra-long intervention cycles, rather than negating the inherent strength-promoting properties of Cr.

### Subgroup analysis by age

In the subgroup analysis stratified by age, discrepancies were observed between the results of the conventional meta-analysis and the RVE sensitivity analysis: the conventional analysis showed that the middle-aged and elderly group had a more significant Cr-induced muscle strength-promoting effect compared with the young group; however, the RVE results indicated that the post-intervention effect size of the young group was not significantly higher than that of the middle-aged and elderly group. This discrepancy likely reveals the existence of data structure issues in the raw data—issues that the conventional model failed to properly address.

Conventional meta-analysis assumes by default that effect sizes are mutually independent, and fails to account for the intrinsic correlation among multiple test indicators within the same study. Furthermore, it also fails to detect the intrinsic heterogeneity in some studies, which exaggerates the contribution of effect sizes with positive effects but high variability in the meta-analysis. For instance, in Candow et al. (2015), the standard deviation (SD) of BP effect data after Cr intervention was as high as 33.6, far exceeding half of its mean value; similar high-variability effects were also observed in studies such as Amiri & Sheikholeslami-Vatani (2023) and Brose, Parise & Tarnopolsky (2003). When these effect sizes are included, while it is possible to obtain the result showing that Cr has a greater effect on the middle-aged and elderly population, the effect of Cr supplementation varies significantly among different middle-aged and elderly individuals.

In contrast, the RVE method can sensitively respond to the structural characteristics of the data itself through its built-in weight adjustment algorithm mechanism. The SCE model within RVE calculates comparable between-study heterogeneity levels for the young group and the middle-aged and elderly group (*τ*-youth = 0.238, *τ*-old = 0.244), which indicates that the dispersion degree of effect sizes across different studies within the two subgroups is similar. In this case, the RVE method ultimately analyzed that the young group had a higher point estimate of effect size compared with the middle-aged and elderly group, and the standard errors of the two groups were comparable—this suggests that there is greater between-study variance within the middle-aged and elderly group. Meanwhile, the RVE analysis provides more robust and conservative effect sizes by downregulating the relative weights of the middle-aged and elderly group. Under the premise of these analyses, the RVE method yielded the result that the young group exhibited a non-significantly greater effect on Cr supplementation compared with the middle-aged and elderly group.

However, it is worth noting that, for the middle-aged and elderly subgroup in the current included studies, both the number of studies and effect sizes were smaller than those of the young subgroup; furthermore, there was greater heterogeneity within the middle-aged and elderly subgroup. These two factors—“relatively smaller sample size” and “greater heterogeneity”—themselves limit the ability of RVE to stably estimate the effect of Cr intervention on muscle strength gain in the middle-aged and elderly population. Furthermore, from a physiological perspective, based on existing studies, more clinical evidence seems to indicate that the middle-aged and elderly population are more sensitive to the muscle strength-promoting effect of Cr.

As a polar hydrophilic molecule, Cr requires specific Na^+^/Cl^−^-dependent transporters for cellular uptake (Ghirardini et al., 2021). In fact, over 90% of cellular Cr uptake occurs *via* the Cr transporter (CRt) (Snow & Murphy, 2001). Older adults have lower Cr stores compared with younger adults (Chilibeck et al., 2017); this lower Cr level may promote CRt function through negative feedback regulatory mechanisms such as inhibiting inhibitory protein synthesis, increasing transporter expression, or reducing transporter protein degradation (Odoom, Kemp & Radda, 1996). Indeed, early studies have shown that when extracellular Cr accumulates to a certain level, intracellular Cr content no longer increases, and the function of CRt in striated muscle may even be downregulated (Loike et al., 1988; Green et al., 1996; Guerrero-Ontiveros & Wallimann, 1998). Inferred from the above mechanisms, the middle-aged and elderly population with lower Cr levels may have greater potential for Cr to promote muscle strength gain compared with young adults.

In summary, we believe this study failed to provide a definitive conclusion regarding the impact of age on Cr-induced muscle strength gain; however, through this analysis, we have at least identified that there are significant individual differences in the middle-aged and elderly population’s responses to Cr. Therefore, more convincing analytical results can be obtained by collecting data from larger-sample studies combined with the RVE sensitivity analysis method, thereby providing a basis for the realization of personalized application of Cr supplementation.

### Subgroup analysis by training level

In subgroup analyses evaluating training background effects, studies involving trained participants exclusively recruited high-level athletes or military personnel engaged in long-term structured training. Compared to untrained individuals, these populations likely exhibit near-physiological ceiling levels of intramuscular Cr due to chronic dietary and exercise adaptations. When endogenous Cr concentrations approach or exceed this physiological threshold, supplemental Cr administration cannot further augment strength outcomes through additional Cr pool expansion (Hultman et al., 1996; Buford et al., 2007).

Both the conventional meta-analysis and RVE sensitivity analysis showed that the untrained population had greater muscle strength gain after Cr supplementation than the trained population. In the RVE analysis, however, the between-group difference in effect sizes did not reach statistical significance, which may be affected by the smaller sample size (five effect sizes) of the untrained group and its high within-group heterogeneity (*τ* = 0.659). Nevertheless, data reveal that the effect size of the untrained group was much larger than that of the trained group (ΔSCE-SMD ≈ 0.63), which means the untrained group derived far greater benefits from Cr supplementation than the trained group.

Both the results of the conventional meta-analysis and the RVE robust variance estimation consistently indicate that the muscle strength-promoting effect of Cr is more significant in the untrained population. However, we also found that the muscle strength-promoting effect of Cr in the untrained population has relatively high within-group heterogeneity, which indicates that different individuals within the untrained population also have differences in their sensitivity to the effect of Cr supplementation. This may provide a direction for subsequent studies, namely: among the same untrained population, which specific individual characteristics affect the effect of Cr on promoting muscle strength gain. In turn, the most suitable population for Cr supplementation can be identified more precisely, thereby providing more accurate usage references for “novices” without professional sports training.

### Subgroup analysis by Cr dosage

Similar to the results of the training level analysis, both the conventional meta-analysis and RVE sensitivity analysis showed that the low-dose group had greater muscle strength gain after Cr supplementation than the high-dose group. This result indicates that the relationship between Cr’s muscle strength-promoting effect and dosage is not a positive linear correlation, which is also consistent with the supplementation effect characteristics of most related supplements, namely: during supplement use, there exists an “optimal dosage”; if the dosage exceeds this “optimal dosage”, the effect may not increase but instead decrease.

As mentioned earlier, the functional realization of Cr supplements is closely related to the transport function of CRt, and this provides further analytical clues for the physiological mechanism by which high-dose Cr is counterproductive to muscle strength gain: high-dose Cr may inhibit the normal function of CRt, thereby reducing the utilization of Cr in muscle cells and ultimately decreasing the muscle strength-promoting effect of Cr. As a member of the solute carrier family, the Cr transporter (CRT) primarily fulfills its transport function through the electrochemical gradient formed by Na^+^ and Cl^−^ (Christie, 2007). Among these ions, Na^+^ is an absolute prerequisite, as it provides the driving force for Cr to enter cells (the role of Cl^−^ in this transport process is not yet clear and is not the focus here) (Chen, Reith & Quick, 2004). During Cr transport, CRT carries Cr along with Na^+^ into the cell (2 Na^+^ molecules are co-transported for every 1 Cr molecule transported). Subsequently, Na^+^/K^+^-ATPase pumps the Na^+^ that has entered the cell out of the cell by consuming energy, thereby maintaining the homeostasis of the transmembrane electrochemical gradient and simultaneously providing a substrate basis for the continuous normal operation of CRT (Dai et al., 1999; Chen, Reith & Quick, 2004; Christie, 2007). Under conditions of high extracellular Cr concentration, a large amount of Na^+^ enters the cell along with the massive influx of Cr. At this point, Na^+^/K^+^-ATPase needs to consume more energy to pump out this additional Na^+^, which increases the intracellular energy metabolism load. When the extracellular Cr concentration exceeds a certain limit, this negative effect on cellular energy metabolism is further amplified, preventing Na^+^/K^+^-ATPase from functioning effectively. This, in turn, impairs the normal function of CRT and ultimately affects the function of Cr in muscle cells.

Interestingly, similar to the effect size results of the training level subgroup analysis, in the dosage subgroup analysis, the point estimate of the effect size for the low-dose group was significantly larger than that for the high-dose group, yet no statistical significance was observed, while high heterogeneity was exhibited (*τ* = 0.864). The lack of statistical significance may also be related to the smaller sample size and greater heterogeneity. Highly consistent results were obtained from the conventional meta-analysis and RVE sensitivity analysis, both of which indicate that low-dose supplementation regimens yield superior muscle strength gain effects—and this finding should not be overlooked. In the future, based on the results of this analysis, the causes of high heterogeneity in the low-dose Cr group can be further explored, so as to provide more precise guidance for the use of related supplements in subsequent practices.

### Subgroup analysis on the impact of training intensity on Cr’s effect

**Figure 16:**
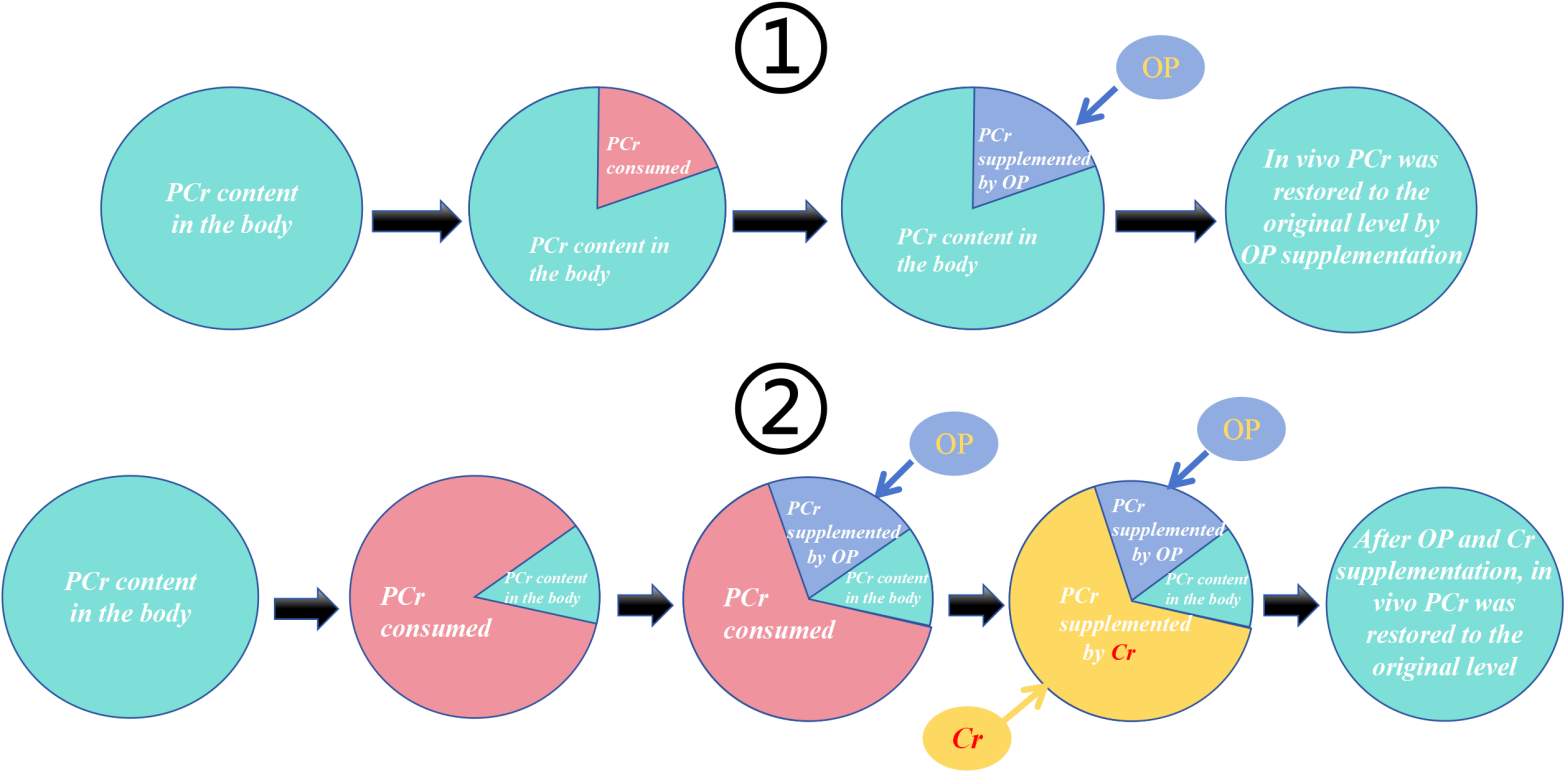
Schematic diagram of the different effects of Cr supplementation during low and high intensity training. ① During low-intensity training, the PCr(red section) consumed by the body can be replenished by the OP (blue section) without any other means of replenishment. ② During high-intensity training, the PCr in the body is almost exhausted(red section), and at this time, it is not enough to rely on OP supplement to completely replenish the consumed PCr(blue section). At this time, Cr ingested from outside the body can help generate PCr(yellow section). Speed up PCr supplement efficiency.

In subgroup analyses of training intensity effects on Cr efficacy, all three studies in the high-intensity training subgroup demonstrated statistically significant post-intervention strength improvements with Cr supplementation. Conversely, within the low-intensity training subgroup, Law et al. (2009)—employing the lowest intensity protocol (60% 1RM)—was the only study showing non-significant strength gains. These findings suggest a positive correlation between training intensity magnitude and Cr-mediated ergogenic potential. Mechanistically, the finite Cr pool reserves in humans are rapidly depleted during high-intensity training, resulting in marked phosphoCr (PCr) depletion. Cr supplementation under such conditions elevates PCr substrate availability, enhances ATP resynthesis efficiency through the Cr kinase reaction, and thereby potentiates strength gains (Fig. 16) (Mujika & Padilla, 1997). Participants in the high-intensity training group were all trained individuals. Due to long-term participation in high-intensity resistance training, this population exhibits a higher proportion of type II muscle fibers, and Cr (Cr) demonstrates greater sensitivity in type II muscle fibers (Johnson et al., 1973).

Notably, the observed heightened sensitivity to Cr in trained individuals does not contradict the previously reported conclusion of greater absolute strength gains in untrained populations, as this apparent paradox stems from two synergistic mechanisms under high-intensity training conditions: rapid depletion of endogenous Cr stores creates enhanced Cr pool expansion capacity, and trained individuals exhibit superior Cr retention efficiency compared to untrained counterparts, thereby enabling more effective utilization of supplemented Cr for strength potentiation. Based on the above, we may conclude that trained individuals under high-intensity training can further augment the effect of Cr in enhancing muscle strength.

### Limitations

Meta-analysis is a secondary analysis of existing studies. Although this analysis not only used the conventional meta-analysis method but also employed the RVE method for sensitivity estimation of effect sizes to enhance the robustness of effect sizes, the conclusions of both the conventional meta-analysis and RVE robust variance estimation depend on the quality of original literature. Thus, they are inevitably limited by the design flaws of original studies (*e.g.*, selection bias, measurement bias), data integrity (*e.g.*, missing outcome data), and publication bias. As this study adopted the meta-analysis method, it inevitably has the aforementioned limitations (LeLorier et al., 1997). Furthermore, during the process of study and data collection, relatively little data on female participants was collected, so we did not include them in this meta-analysis.

### Prospect

This study also intends to outline three critical directions for future research: First, current investigations into optimal dosage and target training intensity remain at the level of phenomenological description, necessitating deeper mechanistic exploration from perspectives such as pharmacokinetics and regulatory mechanisms of skeletal muscle bioenergetic homeostasis. Second, existing evidence demonstrates significant gender bias, with female participants accounting for only a minority of study samples. This requires systematic subgroup analyses to dissect the influences of sex hormone levels and gender-specific differences in body composition on the ergogenic effects of Cr. Finally, this analysis provides preliminary guidance for the use of Cr. However, limited by the quality and flaws of the original studies, the effect sizes of results in certain subgroups exhibit high heterogeneity. Future studies can take the results of this analysis as preliminary guidance to further explore in depth the specific factors affecting Cr’s effect on promoting muscle strength gain, thereby guiding the public in the more precise use of Cr.

## Conclusion

This meta-analysis draws the following conclusions: Cr supplementation exerts a significantly promoting effect on improving muscle strength in the general population, with its effect characteristics exhibiting multidimensional heterogeneity. Specifically, the untrained population shows higher intervention sensitivity compared with the trained population. In terms of the dose–effect relationship, low-dose supplementation regimens demonstrate greater muscle strength improvement benefits than high-dose groups, suggesting the possible existence of nonlinear dose–response characteristics. Meanwhile, there is also a significant synergistic effect between muscle strength gain and training intensity—during the supplementation period, combining with moderate-to-high intensity training can lead to a more significant increase in muscle strength than moderate-to-low intensity training, which provides key evidence-based support for formulating precise intervention regimens. Regrettably, this meta-analysis failed to reach a definitive conclusion regarding the difference in Cr’s effect between the middle-aged and elderly population and the young population; future studies with larger sample sizes are required to conduct more accurate effect size analysis.

## Supplemental Information

10.7717/peerj.20380/supp-1 Supplemental Information 1 The raw data of muscle strength tests, all measured in kilograms (kg) The baseline values and post-intervention values of muscle strength tests for the subjects. All statistical analyses conducted in this meta-analysis were derived from the data.

10.7717/peerj.20380/supp-2 Supplemental Information 2 R code for the Robust Variance Estimation (RVE) analysis method, and results The RVE results of this analysis can be fully replicated using this set of R code and the aforementioned raw data

10.7717/peerj.20380/supp-3 Supplemental Information 3 PRISMA checklist

## Additional Information and Declarations

### Data Availability

The following information was supplied regarding data availability: The raw data is available in the Supplementary File.
