# Supplementary material for: Effects of creatine supplementation on muscle strength gains—a meta-analysis and systematic review
Source: PeerJ. 2025 Nov 27;13:e20380. doi: 10.7717/peerj.20380 (PMC12665265; doi:10.7717/peerj.20380)
Supplement: Supplemental Information 1 — The baseline values and post-intervention values of muscle strength tests for the subjects. All statistical analyses conducted in this meta-analysis were derived from the data. [file peerj-13-20380-s001.docx]

| **STUDY** | **MUSCLE STRENGTH** | | | |
| --- | --- | --- | --- | --- |
|  | **CREATINE GROUP** | | **CONTROL GROUP** | |
|  | **BEFORE** | **AFTER** | **BEFORE** | **AFTER** |
| Amiri E (2023) | 32.15±21.83 | 47±31.53 | 19.62±9.81 | 25.68±14.40 |
| Bonilla DA (2021） | 110.62±13.36 | 132.16±17.72 | 113±16.02 | 121.31±19.87 |
| Brose A (2003) | M:88.076±21.338  F:50.394±12.712 | M:110.776±24.97  F:69.462±24.516 | M:75.364±17.706  F:47.67±11.804 | M:104.874±14.982  F:69.008±13.62 |
| Camic CL(2014) | 88.5±4.0 | 92.8±3.7 | 87.0±4.1 | 89.2±4.1 |
| Candow DG(2015) | 50.0±26.2 | 65.2±33.6 | 49.3±20.2 | 51.2±16.4 |
| Candow DG(2020) | 111±25 | 180±27 | 105±24 | 181±28 |
|  | 97±15 | 108±13 | 92±15 | 114±14 |
| Gualano B(2014) | 33.9±5.6 | 36.5±7.1 | 31.2±7.9 | 33.0±4.9 |
|  | 83.8±19.4 | 97.7±21.7 | 75.5±14.2 | 85.5±13.9 |
| Kaviani M(2019) | 73±9 | 85±2 | 72±4 | 79±6 |
|  | 115±21 | 162±16 | 114±24 | 147±12 |
| Law YL(2009) | 73.75±9.91 | 77.19±10.81 | 68.75±15.70 | 74.38±17.26 |
|  | 99.69±13.26 | 110.94±15.00 | 108.44±19.86 | 109.69±15.00 |
| Samadi M(2022) | 202±16.4 | 205±17.71 | 201.5±20.4 | 204.16±19.47 |
| Stone MH(1999) | 149.7±9.0 | 167±9.5 | 149.8±11.1 | 162.2±10.3 |
|  | 124.5±6.3 | 136.9±6.6 | 129.1±5.6 | 134.1±5.7 |
| Syrotuik DG(2001) | 46.8±14.5 | 49.1±15.5 | 48.2±19.5 | 50.9±20.9 |
| VandenbergheK  (1997) | 25.878±1.362 | 37.682±0.27 | 25.878±1.816 | 32.234±2.724 |
|  | 21.338±0.908 | 30.872±1.816 | 21.792±1.362 | 29.964±1.816 |
| Wang CC(2018) | 133.67±14.07 | 178.33±16.86 | 131.67±15.77 | 165.66±14.62 |
