# Supplementary material for: Effects of creatine supplementation on muscle strength gains—a meta-analysis and systematic review
Source: PeerJ. 2025 Nov 27;13:e20380. doi: 10.7717/peerj.20380 (PMC12665265; doi:10.7717/peerj.20380)

**1.Effect size calculation**

install.packages("metafor")

install.packages("clubSandwich")

library(tidyverse)

install.packages("readxl")

library(readxl)

data <- read_excel("C:/Users/Administrator/Desktop/Rdate.xlsx", sheet = "Sheet1")

colnames(data) <- c("STUDY", "PROJECT", "CrN", "pre_mean_cr", "pre_sd_cr",

"post_mean_cr", "post_sd_cr", "CONN", "pre_mean_con",

"pre_sd_con", "post_mean_con", "post_sd_con")

r <- 0.5

data$change_mean_cr <- data$post_mean_cr - data$pre_mean_cr

data$change_sd_cr <- sqrt(data$pre_sd_cr^2 + data$post_sd_cr^2 - 2 * r * data$pre_sd_cr * data$post_sd_cr)

data$change_mean_con <- data$post_mean_con - data$pre_mean_con

data$change_sd_con <- sqrt(data$pre_sd_con^2 + data$post_sd_con^2 - 2 * r * data$pre_sd_con * data$post_sd_con)

results <- apply(data, 1, function(row) {

n_cr <- as.numeric(row["CrN"])

n_con <- as.numeric(row["CONN"])

m_cr <- as.numeric(row["change_mean_cr"])

m_con <- as.numeric(row["change_mean_con"])

sd_cr <- as.numeric(row["change_sd_cr"])

sd_con <- as.numeric(row["change_sd_con"])

smd <- escalc(measure = "SMD",

m1i = m_cr, m2i = m_con,

sd1i = sd_cr, sd2i = sd_con,

n1i = n_cr, n2i = n_con)

return(c(smd$yi, smd$vi))

})

results_df <- as.data.frame(t(results))

colnames(results_df) <- c("SMD", "Variance")

results_df$SE <- sqrt(results_df$Variance)

final_data <- cbind(data, results_df)

print(final_data[, c("STUDY", "PROJECT", "SMD", "SE")])

###################################################################################

data_with_diff<-raw_data%>%

group_by(STUDY) %>%

mutate(

diff_creatine=post_mean_cr-pre_mean_cr,

sd_diff_creatine = sqrt(pre_sd_cr^2+post_sd_cr^2-2*0.7*pre_sd_cr*post_sd_cr),

diff_control=post_mean_con-pre_mean_con,

sd_diff_control=sqrt(pre_sd_con^2+post_sd_con^2-2*0.7*pre_sd_con*post_sd_con)) %>%

ungroup()

data_with_smd <-data_with_diff%>%

group_by(STUDY)%>%

mutate(S_p=sqrt(((CrN-1)*sd_diff_creatine^2+(CONN-1)*sd_diff_control^2)/

(CrN+CONN-2)),

smd=(diff_creatine-diff_control)/S_p,

se=sqrt(1/CrN+1/CONN+smd^2/(2*(CrN+CONN))),

V=se^2) %>%

ungroup()

select(data_with_smd, STUDY, smd, se, V)


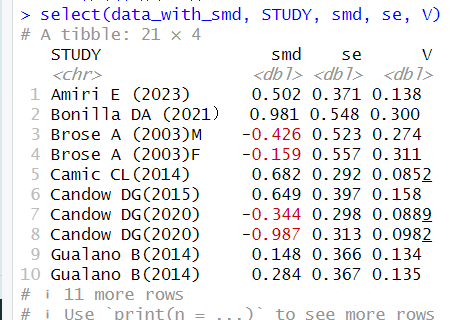


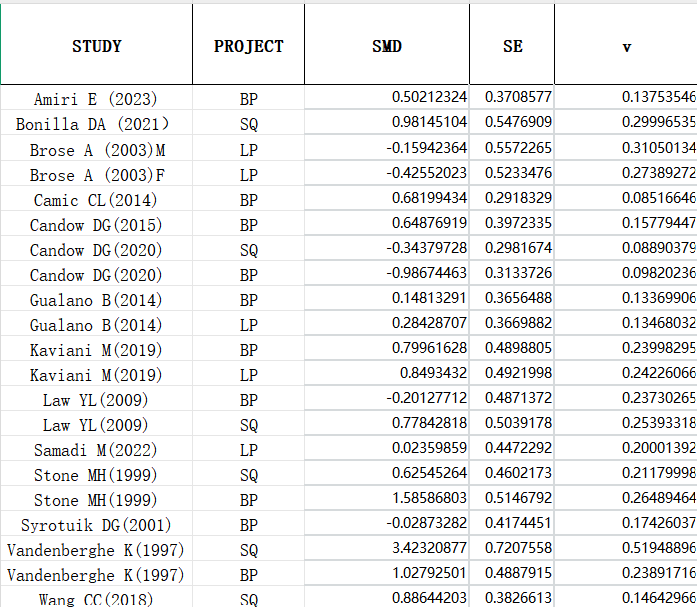


**2.Overall analysis**

library(tidyverse)

library(metafor)

library(clubSandwich)

library(readxl)

library(kableExtra)

data <- read_excel("C:/Users/Administrator/Desktop/Rdate - 长格式.xlsx", sheet = "Sheet1")

head(data)

data <- data %>%

mutate(study_id = as.factor(STUDY), es_id = row_number() )

rho <- 0.6

V_mat <- impute_covariance_matrix(

vi = data$v,

cluster = data$study_id,

r = rho,

smooth_vi = TRUE

)

model <- rma.mv(

yi = SMD,

V = V_mat,

random = ~ 1 | study_id / es_id,

data = data,

method = "REML",

sparse = TRUE

)

summary(model)

robust_ci <- conf_int(model, vcov = "CR2")

robust_ci

tau <- sqrt(model$sigma2[1])

omega <- sqrt(model$sigma2[2])

results_table <- robust_ci %>%

as_tibble(rownames = "Term") %>%

mutate(

Estimate = beta,

`Std. Error` = SE,

`95% CI Lower` = CI_L,

`95% CI Upper` = CI_U,

p_value = ifelse(Term == "intrcpt", model$pval, NA)

) %>%

select(Term, Estimate, `Std. Error`, `95% CI Lower`, `95% CI Upper`, p_value)

vcomp <- tibble(

Term = c("tau", "omega"),

Estimate = c(tau, omega),

`Std. Error` = NA,

`95% CI Lower` = NA,

`95% CI Upper` = NA,

p_value = NA

)

final_table <- bind_rows(results_table, vcomp)

final_table %>%

kable(

digits = 3,

caption = "Overall Meta-Analysis with Robust Variance Estimation (RVE)",

align = "c"

) %>%

kable_styling(full_width = FALSE) %>%

row_spec(1, bold = TRUE) %>%

column_spec(1, bold = TRUE)


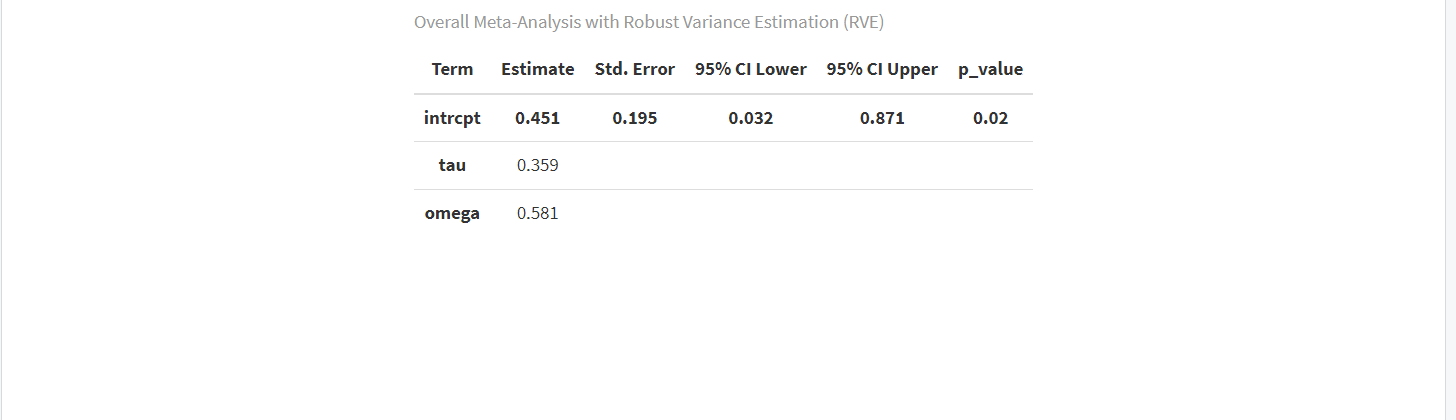


**3.Subgroup Analysis by Age**

library(tidyverse)

library(metafor)

library(clubSandwich)

library(robumeta)

library(readxl)

library(kableExtra)

data <- read_excel("C:/Users/Administrator/Desktop/年龄Rdate - 长格式.xlsx", sheet = "Sheet1")

head(data)

data$age_group <- factor(data$age_group, levels = c("youth", "old"))

data <- data %>%

mutate(esid = row_number())

rho <- 0.6

data$V <- data$v

data <- data %>%

group_by(STUDY) %>%

mutate(studyid = cur_group_id()) %>%

ungroup()

n_studies <- n_distinct(data$studyid)

n_effects <- nrow(data)

cat("Number of studies:", n_studies, "\n")

cat("Number of effect sizes:", n_effects, "\n")

N_age_group <- data %>%

group_by(age_group, studyid) %>%

summarise(effects = n(), .groups = 'drop') %>%

group_by(age_group) %>%

summarise(

studies = n(),

effects = sum(effects)

)

age_robu <- robu(SMD ~ 0 + age_group + SE,

var.eff.size = V,

studynum = studyid,

data = data,

rho = rho)

print(age_robu)

Wald_age_robu <- Wald_test(age_robu,

constraints = constrain_equal(1:2),

vcov = "CR2")

print(Wald_age_robu)

V_mat <- impute_covariance_matrix(data$V,

cluster = data$studyid,

r = rho,

smooth_vi = TRUE)

age_multilevel <- rma.mv(SMD ~ 0 + age_group,

V = V_mat,

random = ~ 1 | studyid / esid,

data = data,

sparse = TRUE)

print(age_multilevel)

CI_age_multilevel <- conf_int(age_multilevel, vcov = "CR2")

print(CI_age_multilevel)

Wald_age_multilevel <- Wald_test(age_multilevel,

constraints = constrain_equal(1:2),

vcov = "CR2")

print(Wald_age_multilevel)

V_age_subgroup <- impute_covariance_matrix(data$V,

cluster = data$studyid,

r = rho,

smooth_vi = TRUE,

subgroup = data$age_group)

age_subgroup <- rma.mv(SMD ~ 0 + age_group,

V = V_age_subgroup,

random = list(~ age_group | studyid),

struct = "DIAG",

data = data,

sparse = TRUE)

print(age_subgroup)

# RVE standard errors

CI_age_subgroup <- conf_int(age_subgroup, vcov = "CR2")

print(CI_age_subgroup)

# Robust F-test

Wald_age_subgroup <- Wald_test(age_subgroup,

constraints = constrain_equal(1:2),

vcov = "CR2")

print(Wald_age_subgroup)

CI_age_robu <- conf_int(age_robu, vcov = "CR2")

vcomp_age_robu <- data.frame(term = "tau",

beta = sqrt(as.numeric(age_robu$mod_info$tau.sq)))

vcomp_age_multilevel <- data.frame(

term = c("tau", "omega"),

beta = sqrt(age_multilevel$sigma2)

)

vcomp_age_subgroup <- data.frame(

term = rownames(CI_age_subgroup)[1:2],

tau = sqrt(age_subgroup$tau2)

)

age_Wald_tests <-

list(

robumeta = Wald_age_robu,

multilevel = Wald_age_multilevel,

subgroup = Wald_age_subgroup

) %>%

map(as_tibble, rownames = "term") %>%

bind_rows(.id = "Model") %>%

select(Model, term, Est = p_val) %>%

mutate(term = "Wald test p-value")

age_results <-

list(

robumeta = bind_rows(as_tibble(CI_age_robu, rownames = "term"), vcomp_age_robu),

multilevel = bind_rows(as_tibble(CI_age_multilevel, rownames = "term"), vcomp_age_multilevel),

subgroup = left_join(as_tibble(CI_age_subgroup, rownames = "term"), vcomp_age_subgroup)

) %>%

bind_rows(.id = "Model") %>%

select(Model, term, Est = beta, SE, tau) %>%

filter(str_detect(term, "^age_group") | term %in% c("tau", "omega")) %>%

mutate(

term = str_remove(term, "^age_group"),

term = ifelse(term == "", "Intercept", term)

) %>%

bind_rows(age_Wald_tests)

age_table <-

age_results %>%

mutate(

Est_SE = if_else(is.na(SE),

formatC(Est, digits = 3, format = "f"),

paste0(formatC(Est, digits = 3, format = "f"),

" [", formatC(SE, digits = 3, format = "f"), "]"))

) %>%

pivot_wider(id_cols = term, names_from = Model, values_from = c(Est, SE, Est_SE, tau)) %>%

left_join(N_age_group, by = c("term" = "age_group"))

options(knitr.kable.NA = " ")

final_table <- age_table %>%

select(term, studies, effects,

Est_SE_robumeta, Est_SE_multilevel, Est_SE_subgroup, tau_subgroup) %>%

kable(

digits = 3,

escape = FALSE,

col.names = c("Age Group", "Studies", "Effect sizes",

"Est. [SE] (CE)", "Est. [SE] (CHE)", "Est. [SE] (SCE)", "tau (SCE)")

) %>%

kable_styling() %>%

add_header_above(c(" " = 3,

"Correlated Effects" = 1,

"Correlated Hierarchical Effects" = 1,

"Sub-group Correlated Effects" = 2))

print(final_table)

save_kable(final_table, file = "C:/Users/Administrator/Desktop/Age_Group_Meta_Analysis_Results.html")


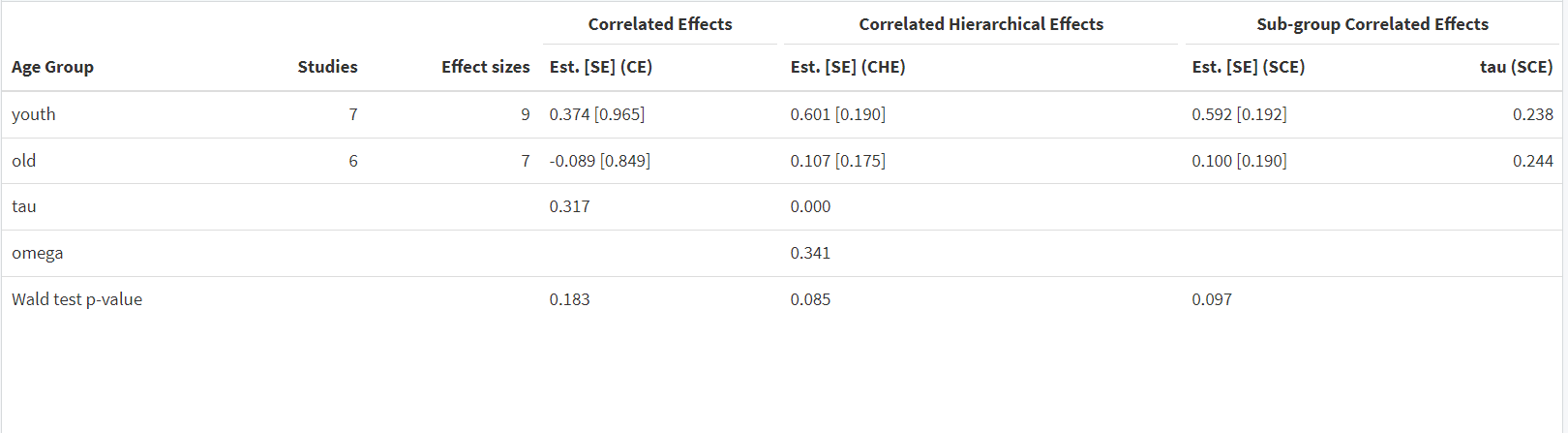


**4.Subgroup Analysis by Training Level**

data <- read_excel("C:/Users/Administrator/Desktop/训练程度Rdate - 长格式.xlsx", sheet = "Sheet1")

head(data)

data$train_group <- factor(data$train_group, levels = c("trained", "untrained"))

data <- data %>%

mutate(esid = row_number())

rho <- 0.6

data$V <- data$v

data <- data %>%

group_by(STUDY) %>%

mutate(studyid = cur_group_id()) %>%

ungroup()

n_studies <- n_distinct(data$studyid)

n_effects <- nrow(data)

cat("Number of studies:", n_studies, "\n")

cat("Number of effect sizes:", n_effects, "\n")

N_train_group <- data %>%

group_by(train_group, studyid) %>%

summarise(effects = n(), .groups = 'drop') %>%

group_by(train_group) %>%

summarise(

studies = n(),

effects = sum(effects)

)

train_robu <- robu(SMD ~ 0 + train_group + SE,

var.eff.size = V,

studynum = studyid,

data = data,

rho = rho)

print(train_robu)

Wald_train_robu <- Wald_test(train_robu,

constraints = constrain_equal(1:2),

vcov = "CR2")

print(Wald_train_robu)

V_mat <- impute_covariance_matrix(data$V,

cluster = data$studyid,

r = rho,

smooth_vi = TRUE)

train_multilevel <- rma.mv(SMD ~ 0 + train_group,

V = V_mat,

random = ~ 1 | studyid / esid,

data = data,

sparse = TRUE)

print(train_multilevel)

CI_train_multilevel <- conf_int(train_multilevel, vcov = "CR2")

print(CI_train_multilevel)

Wald_train_multilevel <- Wald_test(train_multilevel,

constraints = constrain_equal(1:2),

vcov = "CR2")

print(Wald_train_multilevel)

V_train_subgroup <- impute_covariance_matrix(data$V,

cluster = data$studyid,

r = rho,

smooth_vi = TRUE,

subgroup = data$train_group)

train_subgroup <- rma.mv(SMD ~ 0 + train_group,

V = V_train_subgroup,

random = list(~ train_group | studyid),

struct = "DIAG",

data = data,

sparse = TRUE)

print(train_subgroup)

CI_train_subgroup <- conf_int(train_subgroup, vcov = "CR2")

print(CI_train_subgroup)

Wald_train_subgroup <- Wald_test(train_subgroup,

constraints = constrain_equal(1:2),

vcov = "CR2")

print(Wald_train_subgroup)

CI_train_robu <- conf_int(train_robu, vcov = "CR2")

vcomp_train_robu <- data.frame(term = "tau",

beta = sqrt(as.numeric(train_robu$mod_info$tau.sq)))

vcomp_train_multilevel <- data.frame(

term = c("tau", "omega"),

beta = sqrt(train_multilevel$sigma2)

)

vcomp_train_subgroup <- data.frame(

term = rownames(CI_train_subgroup)[1:2],

tau = sqrt(train_subgroup$tau2)

)

train_Wald_tests <-

list(

robumeta = Wald_train_robu,

multilevel = Wald_train_multilevel,

subgroup = Wald_train_subgroup

) %>%

map(as_tibble, rownames = "term") %>%

bind_rows(.id = "Model") %>%

select(Model, term, Est = p_val) %>%

mutate(term = "Wald test p-value")

train_results <-

list(

robumeta = bind_rows(as_tibble(CI_train_robu, rownames = "term"), vcomp_train_robu),

multilevel = bind_rows(as_tibble(CI_train_multilevel, rownames = "term"), vcomp_train_multilevel),

subgroup = left_join(as_tibble(CI_train_subgroup, rownames = "term"), vcomp_train_subgroup)

) %>%

bind_rows(.id = "Model") %>%

select(Model, term, Est = beta, SE, tau) %>%

filter(str_detect(term, "^train_group") | term %in% c("tau", "omega")) %>%

mutate(

term = str_remove(term, "^train_group"),

term = ifelse(term == "", "Intercept", term)

) %>%

bind_rows(train_Wald_tests)

train_table <-

train_results %>%

mutate(

Est_SE = if_else(is.na(SE),

formatC(Est, digits = 3, format = "f"),

paste0(formatC(Est, digits = 3, format = "f"),

" [", formatC(SE, digits = 3, format = "f"), "]"))

) %>%

pivot_wider(id_cols = term, names_from = Model, values_from = c(Est, SE, Est_SE, tau)) %>%

left_join(N_train_group, by = c("term" = "train_group"))

options(knitr.kable.NA = " ")

final_table <- train_table %>%

select(term, studies, effects,

Est_SE_robumeta, Est_SE_multilevel, Est_SE_subgroup, tau_subgroup) %>%

kable(

digits = 3,

escape = FALSE,

col.names = c("train Group", "Studies", "Effect sizes",

"Est. [SE] (CE)", "Est. [SE] (CHE)", "Est. [SE] (SCE)", "tau (SCE)")

) %>%

kable_styling() %>%

add_header_above(c(" " = 3,

"Correlated Effects" = 1,

"Correlated Hierarchical Effects" = 1,

"Sub-group Correlated Effects" = 2))

print(final_table)

save_kable(final_table, file = "C:/Users/Administrator/Desktop/train_Group_Meta_Analysis_Results.html")


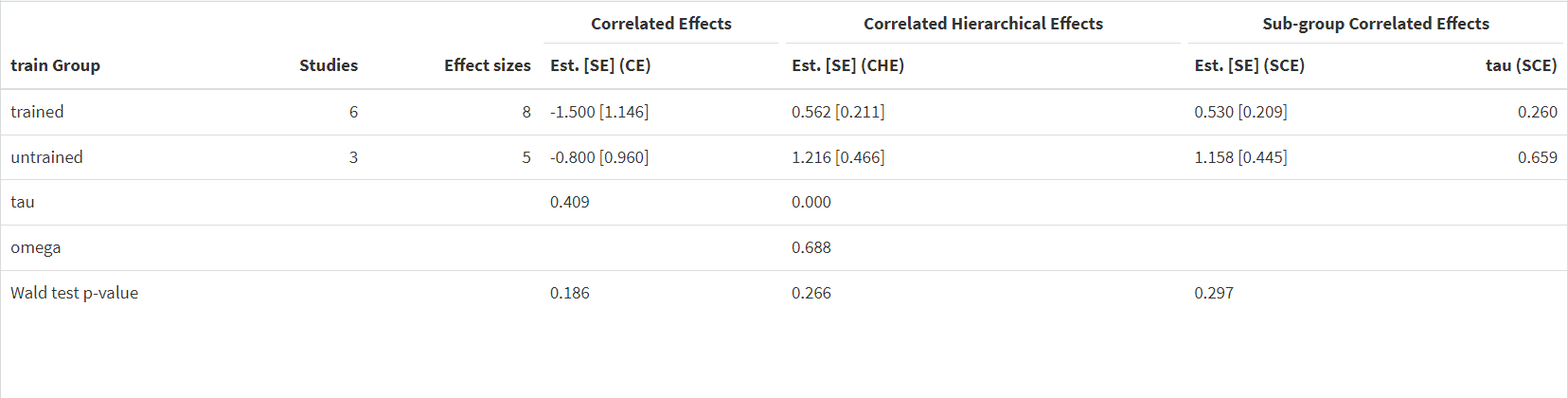


**5.Subgroup Analysis by Creatine Dosage**

data <- read_excel("C:/Users/Administrator/Desktop/剂量Rdate - 长格式.xlsx", sheet = "Sheet1")

head(data)

data$dose_group <- factor(data$dose_group, levels = c("high-dose", "low-dose"))

data <- data %>%

mutate(esid = row_number())

rho <- 0.6

data$V <- data$v

data <- data %>%

group_by(STUDY) %>%

mutate(studyid = cur_group_id()) %>%

ungroup()

n_studies <- n_distinct(data$studyid)

n_effects <- nrow(data)

cat("Number of studies:", n_studies, "\n")

cat("Number of effect sizes:", n_effects, "\n")

N_dose_group <- data %>%

group_by(dose_group, studyid) %>%

summarise(effects = n(), .groups = 'drop') %>%

group_by(dose_group) %>%

summarise(

studies = n(),

effects = sum(effects)

)

dose_robu <- robu(SMD ~ 0 + dose_group + SE,

var.eff.size = V,

studynum = studyid,

data = data,

rho = rho)

print(dose_robu)

Wald_dose_robu <- Wald_test(dose_robu,

constraints = constrain_equal(1:2),

vcov = "CR2")

print(Wald_dose_robu)

V_mat <- impute_covariance_matrix(data$V,

cluster = data$studyid,

r = rho,

smooth_vi = TRUE)

dose_multilevel <- rma.mv(SMD ~ 0 + dose_group,

V = V_mat,

random = ~ 1 | studyid / esid,

data = data,

sparse = TRUE)

print(dose_multilevel)

CI_dose_multilevel <- conf_int(dose_multilevel, vcov = "CR2")

print(CI_dose_multilevel)

Wald_dose_multilevel <- Wald_test(dose_multilevel,

constraints = constrain_equal(1:2),

vcov = "CR2")

print(Wald_dose_multilevel)

V_dose_subgroup <- impute_covariance_matrix(data$V,

cluster = data$studyid,

r = rho,

smooth_vi = TRUE,

subgroup = data$dose_group)

dose_subgroup <- rma.mv(SMD ~ 0 + dose_group,

V = V_dose_subgroup,

random = list(~ dose_group | studyid),

struct = "DIAG",

data = data,

sparse = TRUE)

print(dose_subgroup)

# RVE standard errors

CI_dose_subgroup <- conf_int(dose_subgroup, vcov = "CR2")

print(CI_dose_subgroup)

# Robust F-test

Wald_dose_subgroup <- Wald_test(dose_subgroup,

constraints = constrain_equal(1:2),

vcov = "CR2")

print(Wald_dose_subgroup)

CI_dose_robu <- conf_int(dose_robu, vcov = "CR2")

vcomp_dose_robu <- data.frame(term = "tau",

beta = sqrt(as.numeric(dose_robu$mod_info$tau.sq)))

vcomp_dose_multilevel <- data.frame(

term = c("tau", "omega"),

beta = sqrt(dose_multilevel$sigma2)

)

vcomp_dose_subgroup <- data.frame(

term = rownames(CI_dose_subgroup)[1:2],

tau = sqrt(dose_subgroup$tau2)

)

dose_Wald_tests <-

list(

robumeta = Wald_dose_robu,

multilevel = Wald_dose_multilevel,

subgroup = Wald_dose_subgroup

) %>%

map(as_tibble, rownames = "term") %>%

bind_rows(.id = "Model") %>%

select(Model, term, Est = p_val) %>%

mutate(term = "Wald test p-value")

dose_results <-

list(

robumeta = bind_rows(as_tibble(CI_dose_robu, rownames = "term"), vcomp_dose_robu),

multilevel = bind_rows(as_tibble(CI_dose_multilevel, rownames = "term"), vcomp_dose_multilevel),

subgroup = left_join(as_tibble(CI_dose_subgroup, rownames = "term"), vcomp_dose_subgroup)

) %>%

bind_rows(.id = "Model") %>%

select(Model, term, Est = beta, SE, tau) %>%

filter(str_detect(term, "^dose_group") | term %in% c("tau", "omega")) %>%

mutate(

term = str_remove(term, "^dose_group"),

term = ifelse(term == "", "Intercept", term)

) %>%

bind_rows(dose_Wald_tests)

dose_table <-

dose_results %>%

mutate(

Est_SE = if_else(is.na(SE),

formatC(Est, digits = 3, format = "f"),

paste0(formatC(Est, digits = 3, format = "f"),

" [", formatC(SE, digits = 3, format = "f"), "]"))

) %>%

pivot_wider(id_cols = term, names_from = Model, values_from = c(Est, SE, Est_SE, tau)) %>%

left_join(N_dose_group, by = c("term" = "dose_group"))

options(knitr.kable.NA = " ")

final_table <- dose_table %>%

select(term, studies, effects,

Est_SE_robumeta, Est_SE_multilevel, Est_SE_subgroup, tau_subgroup) %>%

kable(

digits = 3,

escape = FALSE,

col.names = c("dose Group", "Studies", "Effect sizes",

"Est. [SE] (CE)", "Est. [SE] (CHE)", "Est. [SE] (SCE)", "tau (SCE)")

) %>%

kable_styling() %>%

add_header_above(c(" " = 3,

"Correlated Effects" = 1,

"Correlated Hierarchical Effects" = 1,

"Sub-group Correlated Effects" = 2))

print(final_table)

save_kable(final_table, file = "C:/Users/Administrator/Desktop/dose_Group_Meta_Analysis_Results.html")


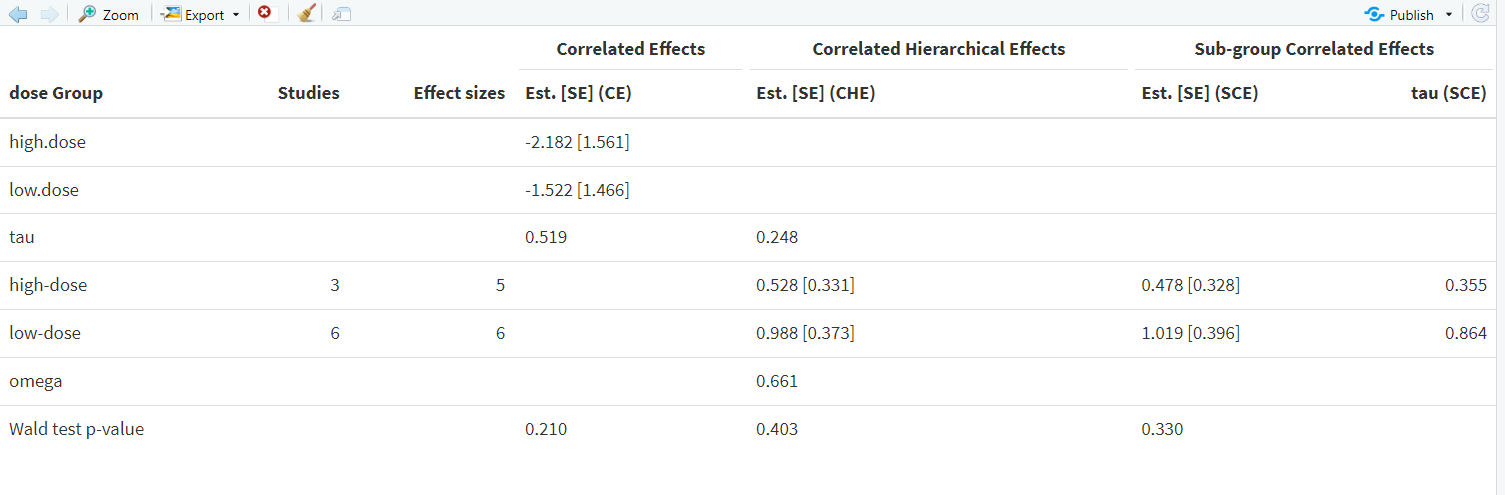

Supplement: Supplemental Information 2 — The RVE results of this analysis can be fully replicated using this set of R code and the aforementioned raw data [file peerj-13-20380-s002.docx]
